# A GATA4-regulated secretory program suppresses tumors through recruitment of cytotoxic CD8 T cells

**DOI:** 10.1038/s41467-021-27731-5

**Published:** 2022-01-11

**Authors:** Rupesh S. Patel, Rodrigo Romero, Emma V. Watson, Anthony C. Liang, Megan Burger, Peter M. K. Westcott, Kim L. Mercer, Roderick T. Bronson, Eric C. Wooten, Arjun Bhutkar, Tyler Jacks, Stephen J. Elledge

**Affiliations:** 1 Division of Genetics, Department of Medicine, Brigham and Women’s Hospital, Boston, MA USA; 2 Department of Genetics, Harvard Medical School, Boston, MA USA; 3 Howard Hughes Medical Institute, Chevy Chase, MD USA; 4 David H. Koch Institute for Integrative Cancer Research, Massachusetts Institute of Technology, Cambridge, MA USA; 5 Department of Biology, Massachusetts Institute of Technology, Cambridge, MA USA; 6 Harvard Medical School, Boston, MA USA; 7 Present Address: Scripps Green Hospital, San Diego, CA USA; 8 Present Address: Human Oncology and Pathogenesis Program, Memorial Sloan Kettering Cancer Center, New York, NY USA

**Keywords:** Immune evasion, Cancer

## Abstract

The GATA4 transcription factor acts as a master regulator of development of multiple tissues. GATA4 also acts in a distinct capacity to control a stress-inducible pro-inflammatory secretory program that is associated with senescence, a potent tumor suppression mechanism, but also operates in non-senescent contexts such as tumorigenesis. This secretory pathway is composed of chemokines, cytokines, growth factors, and proteases. Since GATA4 is deleted or epigenetically silenced in cancer, here we examine the role of GATA4 in tumorigenesis in mouse models through both loss-of-function and overexpression experiments. We find that GATA4 promotes non-cell autonomous tumor suppression in multiple model systems. Mechanistically, we show that *Gata4*-dependent tumor suppression requires cytotoxic CD8 T cells and partially requires the secreted chemokine CCL2. Analysis of transcriptome data in human tumors reveals reduced lymphocyte infiltration in *GATA4*-deficient tumors, consistent with our murine data. Notably, activation of the GATA4-dependent secretory program combined with an anti-PD-1 antibody robustly abrogates tumor growth in vivo.

## Abstract (web summary)

GATA-binding protein 4 (GATA4) is reported to control cell proliferation in cancers. Here the authors show that GATA4’s pro-inflammatory secretome promotes the recruitment of immune cells such as CD8 + T cells to suppress tumour initiation and growth in a non-cell autonomous manner.

## Introduction

Due to homeostatic imbalances that accompany tumorigenesis, cancer cells experience multiple stresses that are detected by a variety of sensory pathways that help ameliorate stress internally and communicate to the rest of the organism that stress is occurring. One such pathway is the DNA damage response (DDR) pathway that is triggered by stresses that act to impact DNA integrity, DNA replication processes, or even the oxidative stress status of cells. One functional output of these sensory pathways is to alert the immune system that unusual events are occurring in case some of the stress-inducing events might involve pathogens. For example, DNA damage has been shown to activate NKG2D ligand expression to bring about innate immune surveillance^1,2^.

Another set of responses activated by the DDR is tumor suppressive in nature and include the activation of apoptosis, which kills cells, and a terminal differentiation pathway called cellular senescence. Senescence is a response to cellular stresses, such as genotoxic stress, shortened telomeres, or oncogenic mutations, which results in an irreversible cell cycle arrest mediated by activation of the CDK inhibitors p21^CIP1/WAF1^ (CDKN1A) via TP53 activation and transcriptional upregulation of p16^INK4A^ (CDKN2A)^1–4^. Furthermore,  this cessation of proliferation cannot be rescued by mitogens and growth factors^3,4^. Senescent cells play a role in physiological processes such as wound healing, but their accumulation in organisms during aging has also been implicated in promoting age-related pathologies such as loss of tissue homeostasis, atherosclerosis, and tau-dependent pathology^5–9^. Consistent with a role in aging^10–13^, properties that regulate the rate of senescence induction, such as telomere length and the rate of telomere attrition, are linked to lifespan^14–19^.

Associated with senescence is an inflammatory secretory response called SASP^5,6^. While senescence-associated, the term SASP is somewhat imprecise in the sense that it happens to be activated by similar stresses but can be activated in replicating cells independently of the cell cycle arrest pathways that are required for senescence. This secretory response to DNA damage can be genetically separated from the mitotic arrest in senescent cells by mutation of the GATA-binding protein 4 (GATA4) transcription factor. GATA4 has established roles in the development of tissues in the heart, testis, liver, foregut, and pancreas^16^. We recently identified GATA4 as the master transcriptional regulator of this damage-induced secretory response and the miR146a gene, previously used to identify genes that contribute to senescence in genetic screens^17,18^. GATA4 protein stability is regulated by selective autophagy via association with p62 (*SQSTM1*). In response to DNA damage and oncogenic stress, GATA4 interaction with p62 is abrogated, resulting in increased GATA4 stability and expression of downstream secreted proteins including chemokines, cytokines, growth factors, and proteases^19,20^. Notably, GATA4 functions independently of the TP53-CDKN1A and RB-CDKN2A pathways but like the TP53 pathway is regulated by USP28^21^ which is a direct target of the DDR-activated ATM protein kinase^22,23^. Activated GATA4 not only positively regulates the NFkB transcription factor but also displays NFkB-independent functions^17^. Previous studies have nominated *GATA4* as a candidate tumor suppressor gene in part because *GATA4* experiences promoter hypermethylation in glioblastoma, ovarian, endometrial, and colorectal carcinomas^24–28^. Moreover, *GATA4* is thought to regulate cellular proliferation in pancreatic, liver, lung, and breast cancers as restoration of *GATA4* expression in cell line models suppressed tumorigenesis to varying degrees, although in some cases decreased cell division was potentially attributed to differentiation programs regulated by GATA4^29–31^. While these studies focused on exploring the role of GATA4 in controlling proliferation, its role as a regulator of the pro-inflammatory secretome in cancer has not been extensively studied in vivo. Therefore, interrogation of *GATA4* provides an opportunity to study the poorly understood role of this stress-regulated secretory response during tumorigenesis. Importantly, mechanisms that bypass senescence induction are required for tumor progression, and GATA4-dependent effects in this context would be independent of cell cycle arrest.

Here, we interrogated the role of GATA4 in tumorigenesis via genetic targeting of *Gata4* in multiple in vivo tumor models to clarify its function during tumorigenesis. We found that GATA4 regulates the expression of a secretory program that suppresses tumorigenesis through the recruitment of CD8 T cells.

## Results

### *Gata4* loss enhances tumor progression in an autochthonous model of *Kras*-driven murine lung adenocarcinoma

**Fig. 1:**
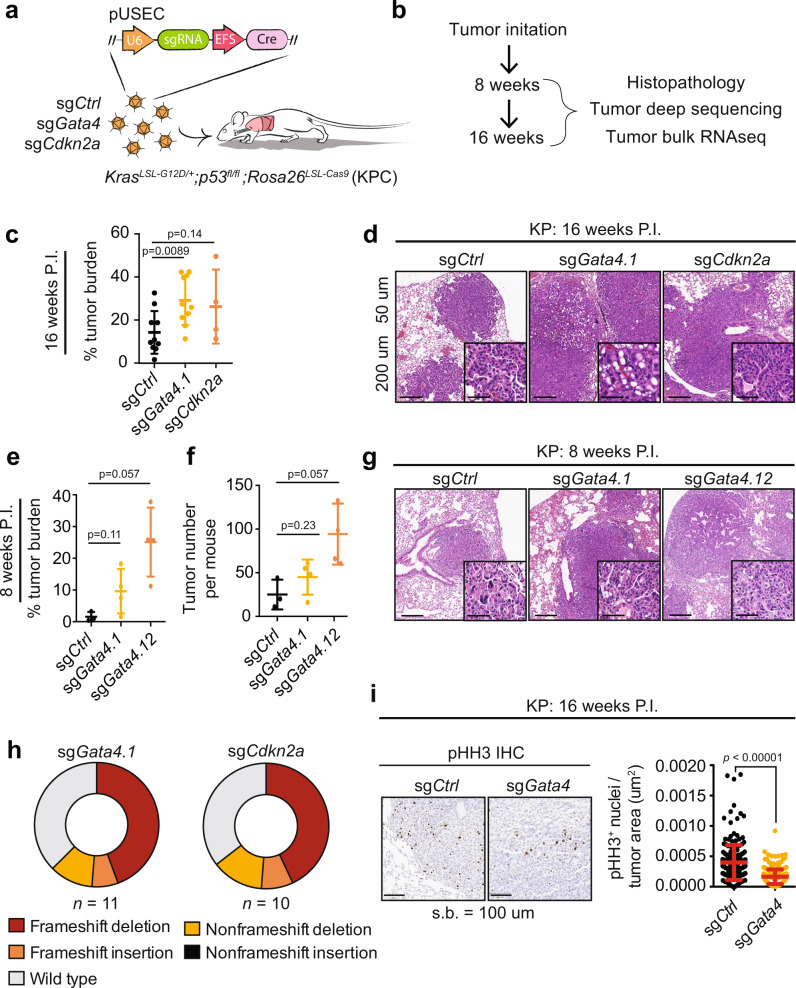
SASP is a tumor suppressor in the KP autochthonous model of lung cancer and affects tumor initiation rather than tumor cell proliferation. **a** Schematic overview of autochthonous lung cancer CRISPR experiment. pUSEC lentiviruses were intratracheally delivered into mouse lungs of *Kras*^*LSL-G12D/+*^;*p53*^*fl/fl*^;*Rosa26*^*LSL-Cas9*^ (KPC) mice to delete genes of interest. sgRNAs include sg*Ctrl* (targeting either *tdTomato* or a safe harbor locus on Chr4.1), sg*Gata4.1*, sg*Gata4.12*, and sg*Cdkn2a*. **b** Tissues were collected 8 and 16 weeks P.I. (post-infection) for histopathology, immunohistochemistry, DNA sequencing, and bulk tumor RNA-seq. **c** Tumor burden at 16 weeks P.I. Burden is defined as total tumor area over total lung area and is assessed from haematoxylin and eosin (H&E) staining of lung sections. Aggregate data from two independent experiments; *n* = 10 for sg*Ctrl* and sg*Gata4* and *n* = 4 for sg*Cdkn2a*. Statistics were derived using a two-sided Mann–Whitney test. **d** Representative H&E images sectioned lung tumor samples at 16 weeks P.I. Error bars represent the mean ∓ s.d. Scale bar is 50 um for larger image and 200 um for inset. The experiment was performed twice independently. **e** Tumor burden at 8 weeks P.I. Burden is defined as in panel **c**. Statistics were derived from a two-sided Mann–Whitney test. Error bars represent the mean ∓ SD. Data are from *n* = 3 mice from sg*Ctrl*, *n* = 4 mice from sg*Gata4.1*, and *n* = 4 mice from sg*Gata4.12*. **f** Tumor number per mouse at 8 weeks P.I. Tumor number is derived by counting individual tumors on H&E stained lung sections. The statistical test is a two-sided Mann–Whitney test. Error bars represent the mean ∓ SD. Data are from *n* = 3 mice from sg*Ctrl*, *n* = 4 mice from sg*Gata4.1*, and *n* = 4 mice from sg*Gata4.12*. **g** Representative images of H&E staining of sectioned tissue samples at 8 weeks P.I. Scale bar is 50 um for larger image and 200 um for inset. The experiment was performed once. **h** CRISPRseq of sgRNA-targeted loci. Sequencing was performed on amplicons centered on the sgRNA cut site. Reads from each biological group were pooled together to show the overall distribution of mutations within each experimental group. Mutational burden was not corrected for tumor purity and therefore is an underestimate of true editing efficiency. Data are from *n* = 11 tumors for sg*Gata4.1* and *n* = 10 tumors for sg*Cdkn2a*. **i** Representative images of IHC staining for pHH3 (phospho-histone H3) (left) and quantification of IHC for pHH3 staining (right) on serial sections of lung tumor tissue at 16 weeks P.I. Statistics were derived from a two-sided Mann–Whitney test. Error bars represent the mean ∓ SD. Data are from *n* = 4 mice totalling 209 tumors for sg*Ctrl* and *n* = 6 mice totalling 327 tumors for sg*Gata4*. Experiment performed once.

TP53 has previously been shown to be a negative regulator of the secretory pathway co-activated along with senescence^19,32^. Given the prevalence of defects in the TP53 pathway in cancer, we chose to explore the role of *Gata4* in tumorigenesis by deleting *Gata4* in a p53-deficient genetically engineered mouse model (GEMM) of lung adenocarcinoma. The KP (*loxP-Stop-lox Kras*^*G12D/+*^*; p53*^*fl/fl*^) GEMM develops lung adenocarcinomas after intratracheal delivery of lentiviral vectors expressing Cre recombinase^33,34^. To rapidly interrogate the functional consequences of *Gata4* loss during tumor initiation in the KP model, we crossed KP mice with a conditional allele of the CRISPR-associated endonuclease, *Cas9* (*Rosa26*^*LSL-Cas9*^; hereafter KPC)^35^. We induced tumors in KPC mice using pUSEC lentiviruses^36^ expressing Cre recombinase and either control sgRNAs (sg*Ctrl*), two independent sgRNAs targeting *Gata4* (sg*Gata4*), or an sgRNA targeting the senescence cell cycle regulator *Cdkn2a* (sg*Cdkn2a*) (Fig. 1a). Lungs were harvested at 8 weeks and 16 weeks post tumor initiation to assess differences in tumor burden and histopathological changes upon targeted somatic genome editing (Fig. 1b). In two independent experiments, mice targeted with sg*Gata4* exhibited increased tumor burden at 16 weeks when compared to sg*Ctrl* mice, suggesting a *Gata4*-dependent tumor suppressive phenotype (Fig. 1c). As expected, somatic disruption of the known senescence cell cycle regulator and potent tumor suppressor, *Cdkn2a,* also resulted in increased tumor burden. *Gata4*-targeted mice had histopathologically more advanced tumors compared to sg*Ctrl* mice, indicating that targeted *Gata4* somatic editing results in more aggressive tumors (Fig. 1d, Supplementary Fig. 1a). Interestingly, analysis of mice 8 weeks post-infection similarly revealed that somatic editing of *Gata4* not only increased both the tumor burden and histopathological grade, but also total tumor numbers when compared to sg*Ctrl* mice (Fig. 1e, f, g, Supplementary Fig. 1b), indicating that *Gata4* loss may contribute to enhanced tumor initiation. Deep sequencing of the *Gata4* locus in micro-dissected tumors revealed a clear selection for loss of function mutations in sg*Gata4* tumors, as was observed for the *Cdkn2a* locus in tumors isolated from sg*Cdkn2a* mice (Fig. 1h, Supplementary Fig. 1c, d). The finding that *Gata4* deficiency impacted both tumor burden and tumor initiation are consistent with the hypotheses that *Gata4* might operate by regulating cellular proliferation to restrict tumor growth. However, immunohistochemistry of phospho-Histone H3 (pHH3), a marker of proliferation, was reduced in sg*Gata4*-targeted tumors when compared to sg*Ctrl* mice at the 16-week timepoint and trended downward at the 8-week timepoint, suggesting overall that an increase in tumor burden in sg*Gata4* mice was not due to an increase in cell cycle activity (Fig. 1i, Supplementary Fig. 1e). Taken together, these data suggest that inactivation of the SASP regulator *Gata4* enhances tumorigenesis by increasing tumor initiation rather than by increasing cell proliferation.

### sg*Gata4*-targeted tumors in the autochthonous model of lung adenocarcinoma have fewer TILs than control tumors

**Fig. 2:**
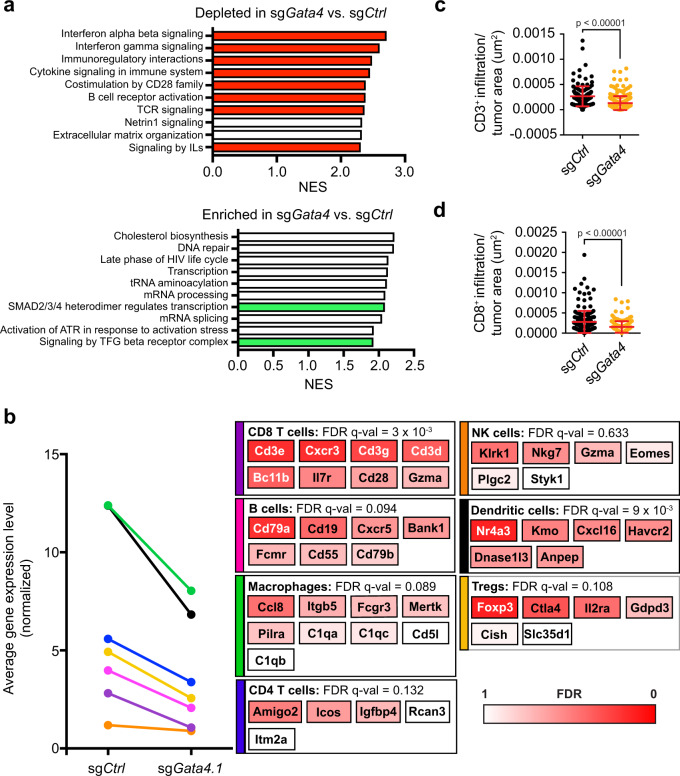
Loss of *Gata4* in vivo is associated with reduction of inflammatory pathways and results in reduced tumor-infiltrating lymphocytes. **a** RNA-seq was performed on bulk tumors and included both tumor and stromal cells. Individual tumors were micro-dissected from mouse lung tissue. GSEA on differentially expressed genes between sg*Gata4*-targeted tumors and sg*Ctrl-*targeted tumors is shown; genesets of interest were derived from the curated genesets from the Reactome database. Genesets of interest that were depleted in sg*Gata4*-targeted tumors versus sg*Ctrl*-targeted tumors are shown in red. Genesets of interest, which were enriched in sg*Gata4*-targeted tumors versus sg*Ctrl*-targeted tumors are shown in green; 10 out of the top 16 differentially expressed genesets are shown. All genesets displayed have a statistically significant FDR < 0.01. **b** Sets of genes whose expression correlates with the abundance of different subsets of immune cells (see Supplementary Data 1) were analyzed for their expression in sg*Gata4*-targeted tumors versus sg*Ctrl*-targeted tumors collected 16 weeks P.I. from the autochthonous KPC lung cancer model. Representative genes characterizing each immune cell type are shown with a heatmap representing the corresponding FDR q-value derived from the RNA-seq analysis of tumors. **c** Quantification of IHC of CD3 staining of serially sectioned lung lobes collected 16 weeks P.I. Error bars represent the mean ∓ SD and statistics derived from two-sided *t*-test. Data are derived from *n* = 4 mice totalling *n* = 139 tumors for sg*Ctrl* and *n* = 6 mice totalling  *n* = 262 tumors for sg*Gata4*. **d** Quantification of IHC of CD8 staining of serially sectioned lung lobes collected 16 weeks P.I. Error bars represent the mean ∓ SD, and statistics were derived from a two-sided *t*-test. Data are derived from *n* = 4 mice totalling *n* = 183 tumors for sg*Ctrl* and *n* = 6 mice totalling *n* = 171 tumors for sg*Gata4*.

We interrogated the transcriptional consequences of CRISPR targeting of *Gata4* by performing RNA-seq on micro-dissected tumors from sg*Gata4*- and sg*Ctrl*-targeted tumor-bearing mice. RNA-seq of bulk tumors revealed a number of differentially expressed genes between *Gata4*-targeted tumors and controls (Fig. 2a-b, Supplementary Data 1). Geneset enrichment analysis (GSEA) on the list of differentially expressed genes showed that pathways depleted in *Gata4*-targeted tumors relative to controls include inflammatory pathways, such as interferon-beta signaling, interferon-gamma signaling, immunoregulatory interactions, cytokine signaling in the immune system, and signaling by interleukins; collectively, these inflammatory pathways are relevant to lymphocytes (Fig. 2a, Supplementary Data 2)^37^. By contrast, enriched pathways in sg*Gata4*-targeted tumors included functions found in metabolically active cells, including cholesterol biosynthesis, DNA repair, transcription, and mRNA processing (Fig. 2a, Supplementary Data 2). Cell cycle and mitotic pathways were not included in the top differentially expressed pathways, again suggesting that cellular proliferation is not the primary driver of *Gata4*-dependent tumor suppression. Among the most enriched pathways expressed in sg*Gata4*-targeted tumors were the TGF beta and SMAD signaling genesets. Activation of the TGF beta-SMAD pathway promotes a potent immunosuppressive environment required for the differentiation, maintenance, and effector functions of suppressive immune populations such as regulatory T cells and myeloid-derived suppressor cells, suggesting that sg*Gata4*-targeted tumors have increased immune-suppressive signaling. Genes whose expression has been previously validated to correlate with the abundance of lymphocytes such as B cells and T cells are also downregulated in sg*Gata4*-targeted tumors, suggesting that sg*Gata4*-targeted tumors experience reduced immune surveillance relative to sg*Ctrl* tumors (Fig. 2b, Supplementary Data 3), and this was confirmed by immunohistochemistry (IHC) for the pan T cell marker CD3 and the cytotoxic T-cell-specific marker CD8 (Fig. 2c-d, Supplementary Fig. 2a). We confirmed that lymphocyte-specific signatures were reduced in tumors isolated from sg*Gata4*-targeted mice by applying genesets derived from published datasets of single-cell RNA sequencing experiments of lymphocytes (Supplementary Fig. 2b, Supplementary Data 3). The phenotype of reduced TILs is specific to sg*Gata4*-targeted tumors and is not present in tumors isolated from sg*Cdkn2a-*targeted *mice* (Supplementary Fig. 3a). Interestingly, the expression of genes required for antigen presentation, such as the interferon-inducible *Tap1* and *Tap2*, are also reduced in tumors from sg*Gata4*-targeted mice (Supplementary Fig. 3b), possibly due to the absence of γ−interferon from TILs. Our findings demonstrate that *Gata4* is important for lymphocyte recruitment to tumors.

### *GATA4* copy number loss is associated with lower immune infiltrate in human tumors

**Fig. 3:**
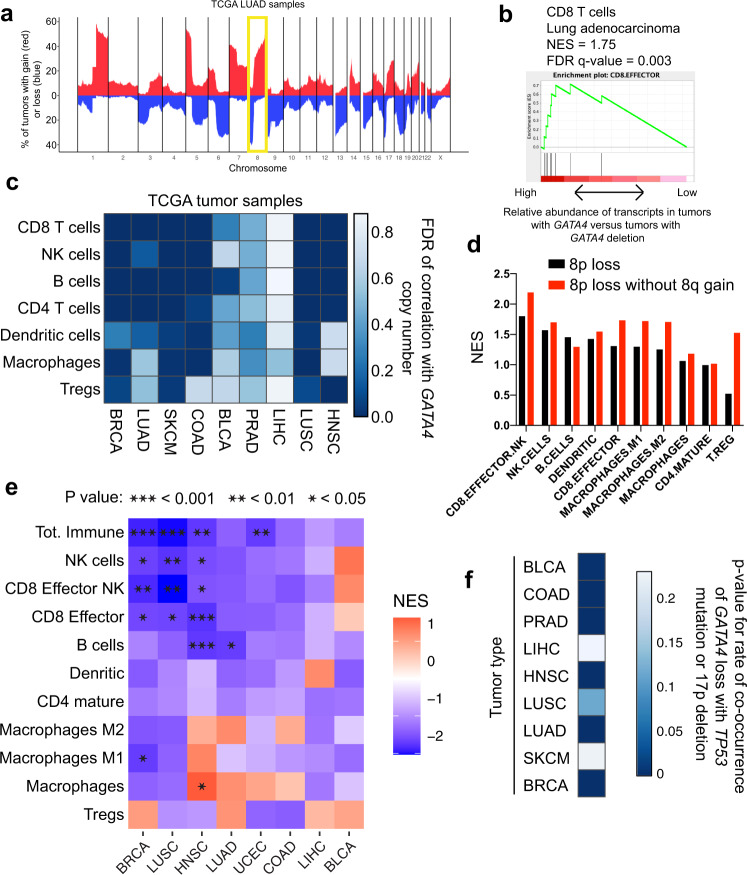
*GATA4* is deleted in human cancer and is associated with the abundance of TILs. **a** Summary of TCGA data showing the most common chromosomal deletions (blue) and amplifications (red) in human lung adenocarcinoma (LUAD) tumor samples. Chromosome arm 8p contains the human *GATA4* gene. Chromosome 8 is highlighted. **b** CD8 T cell-associated transcripts are decreased in *GATA4*-deleted tumors in lung adenocarcinoma. **c** Analysis of the abundance of tumor-infiltrating lymphocytes (TILs) in TCGA tumor samples of various subtypes. Tumor samples were corrected for tumor purity before calling copy number alterations. TCGA RNA-seq data were used to calculate the association between *GATA4* copy number and the abundance of TIL subtypes in each tumor type. Color indicates the FDR of the correlation between *GATA4* copy number and the abundance of TIL subtypes. Darker colors indicate higher significance. Abbreviations are breast adenocarcinoma (BRCA; *n* = 256 *GATA4* loss, *n* = 460 no *GATA4* loss), lung adenocarcinoma (LUAD; *n* = 120 *GATA4* loss, *n* = 319 no *GATA4* loss), melanoma (SKCM; *n* = 20 *GATA4* loss, *n* = 82 no *GATA4* loss), colorectal adenocarcinoma (COAD; *n* = 97 *GATA4* loss, *n* = 167 no *GATA4* loss), bladder adenocarcinoma (BLCA; *n* = 138 *GATA4* loss, *n* = 262 no *GATA4* loss), prostate adenocarcinoma (PRAD; *n* = 147 *GATA4* loss, *n* = 266 no *GATA4* loss), liver hepatocellular carcinoma (LIHC; *n* = 216 *GATA4* loss, *n* = 149 no *GATA4* loss), lung squamous cell carcinoma (LUSC; *n* = 124 *GATA4* loss, *n* = 229 no *GATA4* loss), and head and neck squamous cell carcinoma (HNSC; *n* = 148 *GATA4* loss, *n* = 363 no *GATA4* loss). **d** Analysis of the association of loss of *GATA4*/8p with TIL subtypes in samples with or without the co-associated CNA + 8q in colorectal adenocarcinoma. **e** Extended CNA profile normalization analysis across tissue types with respect to TIL subtype abundance associations. Here we normalized tumor cohorts for all *GATA4*//8p-correlated and anticorrelated CNAs in each subtype, beyond just the +8q co-association. Abbreviations are breast adenocarcinoma (BRCA; *n* = 127 *GATA4* loss, *n* = 313 no *GATA4* loss), lung adenocarcinoma (LUAD; *n* = 63 *GATA4* loss, *n* = 277 no *GATA4* loss), uterine corpus endometrial carcinoma (UCEC; *n* = 19 *GATA4* loss, *n* = 92 no *GATA4* loss), colorectal adenocarcinoma (COAD; *n* = 47 *GATA4* loss, *n* = 131 no *GATA4* loss), bladder adenocarcinoma (BLCA; *n* = 68 *GATA4* loss, *n* = 202 no *GATA4* loss), liver hepatocellular carcinoma (LIHC; *n* = 148 *GATA4* loss, *n* = 114 no *GATA4* loss), lung squamous cell carcinoma (LUSC; *n* = 60 *GATA4* loss, *n* = 200 no *GATA4* loss), and head and neck squamous cell carcinoma (HNSC; *n* = 73 *GATA4* loss, *n* = 323 no *GATA4* loss). **f** *GATA4* copy number loss is co-associated with *TP53* loss (mutation or deletion) in most tumor types. *P*-values are calculated using hypergeometric tests.

*GATA4* is not frequently mutated but is frequently deleted in multiple human tumor types and frequently silenced in lung and gastric cancers^28,38^ and 61% of colon cancers^28^. To further explore this, we examined whether genomic alterations affecting *GATA4* correlated with immune infiltration in tumors. *GATA4* resides on chromosome 8p, which is deleted in multiple tumor types at a frequency similar to that of *TP53* deletion on 17p (Fig. 3a, Supplementary Fig. 4a-b). At least one copy of *GATA4*/8p is deleted in 51% of lung adenocarcinoma tumors (Supplementary Figs. 4c, 5c). Based on the immune-cold phenotype observed with targeted loss of *Gata4* in the KPC model, we hypothesized that loss of *GATA4*/8p may correlate with TIL abundance in human tumors. To examine this relationship, we used purity-corrected copy number data from the TCGA database to identify tumors that had lost at least 1 copy of *GATA4* and assessed differential expression in the *GATA4*-deleted tumors compared to *GATA4*-wild-type tumors. We then performed geneset enrichment analysis using immune cell-type-specific genesets to determine whether any immune cell populations were depleted or enriched in *GATA4*-deleted tumors. In six of nine tumor types examined, loss of *GATA4* was significantly associated with decreased abundance of specific immune-related transcript sets indicative of tumor-infiltrating cytotoxic CD8 T cells, CD4 T cells, and B cells (Fig. 3b-c, Supplementary Fig. 6a-b, Supplementary Data 3-4).

Since there are several potentially confounding genomic covariates associated with *GATA4* loss that may affect immune infiltration, including total aneuploidy levels which have been shown to negatively correlate with immune infiltrate (Davoli et al., 2017), as well as specific CNAs such as +8q, which is enriched in −8p samples, we took several different approaches to account for covariates. First, we analyzed the association of *GATA4* loss with immune infiltration in subsets of tumors with overall low levels of aneuploidy, and find that this association is still present (Supplementary Fig. 6c). Second, since the top CNA covariate of loss of 8p is gain of 8q across tumor types (Supplementary Fig. 5b, c), possibly due to structural mechanisms like isochromosome formation, we specifically addressed the +8q covariate, particularly since 8q contains the prominent oncogene *MYC*. We analyzed tumors that lost 8p but did not gain 8q and found that 8p loss in the absence of 8q gain is still associated with lower levels of TIL-associated transcripts (Fig. 3d, Supplementary Fig. 7a, Supplementary Data 5). Thus, a break in the centromeric region of chromosome 8 followed by isochromosome-8q formation or other types of resolution that could facilitate an extra copy of 8q concurrent with 8p loss could have two independent pro-tumorigenic effects: gain of *MYC* to promote cell proliferation and growth, and loss of *GATA4* to reduce immune surveillance.

Finally, to further clarify the association of *GATA4* gene dosage with immune infiltration and to disambiguate it from other chromosome arm-level events that co-occur with *GATA4*/8p loss (not limited to +8q), we developed a cohort CNA normalization strategy, C-Norm, for Copy number covariate Normalization, to control for all correlated and anticorrelated CNAs. We first calculated the over- or under-representation of every arm-level CNA with *GATA4*/8p loss compared to *GATA4*/8p-neutral tumors, then we prioritized samples to mask from either group based on how many over-represented CNAs were present in the sample. After removing a small number of prioritized samples at random, CNA rates were recalculated and this process was repeated iteratively until equal or near-equal incidence of each CNA was achieved in each group, except for the group-defining CNA of interest (*GATA4*/8p loss) (Supplementary Fig. 7b). Most tissue types were compatible with this analysis strategy. We find that the negative associations between *GATA4* loss and immune infiltration are significant in 5 out of 8 tissue types when normalizing all other correlated and anticorrelated copy number events: breast invasive carcinoma, head and neck squamous cell carcinoma, lung squamous cell carcinoma, lung adenocarcinoma, and uterine corpus endometrial carcinoma (Fig. 3e).

SASP has been shown to be negatively regulated by TP53^32^. Loss of *TP53* enhances senescence-associated secretory phenotype induced by DNA damage and would therefore increase the selective pressure for cells to avoid the immune system by reducing SASP. If true, *TP53*-mutant tumors should have co-mutation of *GATA4* more frequently than expected. Upon examination, we found that *GATA4* deletion co-occurs with *TP53* mutation or deletion more often than expected by chance (Fig. 3f) and is more strongly associated with *TP53* loss than other common tissue-specific arm-level losses (Supplementary Fig. 5a).

### *Gata4* is tumor suppressive in multiple cell types

**Fig. 4:**
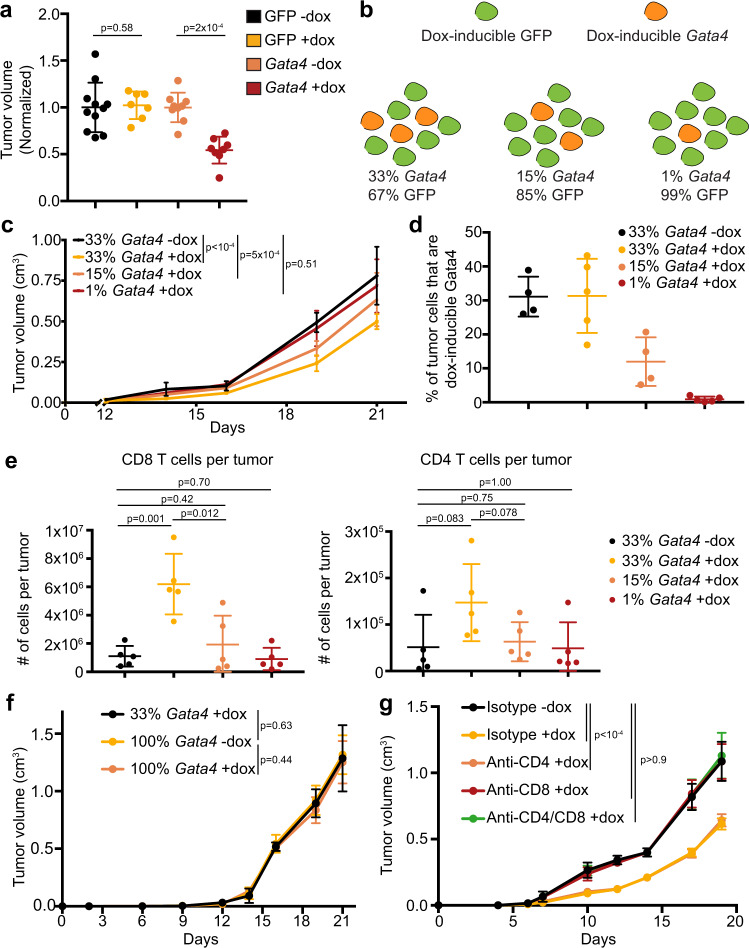
*Gata4* acts in a non-cell-autonomous, CD8 cytotoxic T cell-dependent mechanism to suppress tumor growth. **a** Tumor volumes from experiments. GFP^+^ KP lung cells with either dox-inducible GFP or dox-inducible *Gata4* were transplanted subcutaneously into the flanks of C57BL/6J mice. GFP was expressed in all cell types to control for the potential immunogenicity of GFP. Dox administration began the day before tumor cell transplantation. The experiment was performed twice and the combined normalized volumes from both experiments are displayed. Data are mean ± SD and *n* = 7 tumors for GFP-dox, *n* = 7 tumors for GFP + dox, *n* = 9 tumors for Gata4-dox, and *n* = 8 tumors for Gata4+dox,. *P*-values are from two-tailed Mann–Whitney tests and *n* = 11 for GFP − dox, *n* = 7 for GFP + dox, *n* = 9 for *Gata4* -dox, and *n* = 8 for *Gata4* + dox, where *n* represents the total number of tumors. **b** Schematic overview of the experiment to determine whether *Gata4* has cell-autonomous or non-cell-autonomous effects on tumor suppression and whether cell cycle regulation is important. KP cells were transduced with pInducer30 Gata4-HA (Thy1.1^+^ and GFP^+^). Control KP cells were transduced with pInducer30 GFP-HA (Thy1.1^−^ and GFP^+^). The cell populations are mixed together at the specified ratios and transplanted into immune-competent C57BL/6J mice. Tumor cells can be flow cytometrically differentiated from non-tumor cells via GFP expression. **c** Populations outlined in **b** are mixed with the specified proportions of KP cells with dox-inducible GFP and KP cells with dox-inducible *Gata4*. Cells were transplanted into the flanks of immune-competent C57BL/6J mice; dox was started in the indicated groups one day before tumor cell transplant and administered continuously until endpoint. Tumor volumes throughout the course of the experiment are shown. Data are mean ± SD. *P*-values are from two-tailed Mann–Whitney test at the day 19 timepoint and *n* = 10 mice per group. **d** Flow cytometry-based quantification of the proportion of dox-inducible *Gata4* tumor cells (GFP^+^Thy1.1^+^) as a fraction of all tumor cells (GFP^+^) at the experimental endpoint of the experiment shown in **c**. Data are mean ± SD and *n* = 4 or 5 tumors per group. **e** Flow cytometry-based immunoprofiling of TILs in tumors from the experiment shown in **c**. Tumors were extracted at the experimental endpoint, dissociated to a single-cell suspension, and stained for the indicated TIL subtype. Data shown are quantification of the number of cells of the indicated TIL subtype per tumor. *P*-values are from unpaired two-way *t*-tests and *n* = 5 per group. **f** KP cells with dox-inducible *Gata4* at the specified ratios were transplanted into *Rag1*^−/−^ C57BL/6J mice. The 33% Gata4 population was composed of 33% of cells with dox-inducible *Gata4* and 67% of cells with dox-inducible GFP as in **c**. Dox was administered to mice one day before tumor cell transplant and was administered continuously until endpoint. Tumor volumes are shown. Data shown are mean ± SD and *n* = 10 per group combined from 2 independent experiments. *P*-values are from a two-tailed Mann–Whitney test at the Day 21 timepoint. **g** C57BL/6J mice received depleting antibodies against CD4^+^, CD8^+^, CD4^+^ and CD8^+^ T cells, or an isotype control antibody. All mice received the same total amount of antibody. The single depletion groups received isotype control antibody in addition to depletion antibody in order to ensure that all groups of mice received the same amount of antibody as the double depletion group. KP cells with dox-inducible *Gata4* were transplanted into the flanks of the pre-depleted mice. Dox was initiated one day before transplant in the indicated groups and was administered continuously until the experimental endpoint. Tumor volumes are shown. Data are mean ± SD and *n* = 10 per group combined from 2 independent experiments. *P*-values are from two-tailed Mann–Whitney tests at Day 19 timepoint. Specifically, *p*-values are as follows: *p* < 0.0001 for isotype −dox versus isotype +dox, *p* < 0.0001 for isotype -dox versus anti-CD4 + dox, *p* = 0.9243 for isotype −dox versus anti-CD8 + dox, and *p* = 0.9425 for isotype −dox versus anti-CD4/CD8 + dox. For this figure, source data are provided as a Source Data file.

To develop a tractable system to study the effects of GATA4 on tumor formation, we chose to ectopically activate the *GATA4*-regulated secretory program by forced *Gata4* expression. We developed lentiviral vectors to induce the expression of mouse *Gata4* (hereafter tet-Gata4) or a control cDNA encoding *GFP* (hereafter tet-GFP) and infected B16 murine melanoma cells, PyMT S2WTP3 murine breast cancer cells, and KP-derived primary murine lung adenocarcinoma cells (Supplementary Fig. 8a). With respect to the KP line, we enhanced its immunogenicity by infection with a Lenti-LucOS luciferase fusion construct containing strongly immunogenic CD4 and CD8 model antigens^39–41^. Transplant experiments showed that *Gata4* expression restricted tumor growth and resulted in reduced tumor volume, indicating that *Gata4*’s tumor-suppressive effects are conserved across a variety of tumor types with varying immunogenicity (Fig. 4a, Supplementary Fig. 8b-d).

### *Gata4*’s effects are non-cell-autonomous, dose-dependent, and require cytotoxic CD8 T cells

To investigate whether the tumor-suppressive effects of *Gata4* are cell-autonomous or non-cell-autonomous, we mixed KP lung adenocarcinoma cells containing either tet-Gata4 or tet-GFP. To control for GFP-specific immunogenicity, both the tet-Gata4 and tet-GFP cell populations expressed constitutive GFP. We created a 33% tet-Gata4 and 67% tet-GFP tumor cell population and transplanted the mixture subcutaneously. Upon doxycycline induction, *Gata4* expression in the mixed population led to robust suppression of tumor growth compared to the vehicle-treated group (Supplementary Fig. 8e), indicating that *Gata4* regulates tumor growth via a non-cell-autonomous mechanism. To determine if the effects of *Gata4* on tumor suppression were dose-dependent, we generated mixed populations with the following percentages of tet-Gata4 cells, with the remaining cells being tet-GFP: 33%, 15%, and 1% (Fig. 4b). We transplanted these tumor cell mixtures into the flanks of immunocompetent syngeneic hosts. Consistent with our previous mixing experiment, *Gata4* induction led to decreased tumor size (Fig. 4c). Moreover, tumor volume was inversely proportional to the percent of cells with dox-inducible *Gata4* (Fig. 4c). Flow cytometric analysis of the tumors showed that the percent of tet-Gata4 cells in each tumor remained constant throughout the experiment rather than depleting relative to tet-GFP cells (Fig. 4d). Infiltrating CD45^+^CD3^+^CD4^+^ and CD45^+^CD3^+^CD8^+^ T cells were more abundant in *Gata4-*activated tumors than controls, and the increase was dose-dependent, suggesting that there is a minimum number of *Gata4*-expressing cells necessary in a tumor to achieve maximal tumor suppression (Fig. 4e). Combining the observation that the fraction of *Gata4*-expressing cells did not decrease during tumor growth with the finding that a subset of tumor cells overexpressing *Gata4* is sufficient to suppress tumor growth, suggests that, in this context, *Gata4* promotes tumor control via non-cell-autonomous effects rather than cell-autonomous cell cycle effects.

To determine whether this non-cell-autonomous effect is mediated by the adaptive branch of the immune system, we transplanted tet-Gata4 cells as either a pure or a mixed population into *Rag1*^*−/−*^ syngeneic hosts that lack functional T and B cell compartments. We repeated the tumor cell mixing experiment described above to see whether *Gata4*-mediated tumor suppression depends on adaptive immune responses rather than direct tumor cell-specific paracrine effects. Remarkably, we observed that *Gata4*-mediated tumor suppression was circumvented by loss of the adaptive immune system (Fig. 4f, Supplementary Fig. 9e). Non-adaptive immune mechanisms such as macrophage or NK cell recognition have been shown to clear senescent tumor cells^42–44^, but given that we see no tumor-suppressive effect in *Rag1*^*−/−*^ mice that have macrophages and NK cells, we do not believe there is an obvious role for NK cells in our tumor models.

To unravel which cellular components of adaptive immunity are required, we used CD4- and CD8a-specific depleting antibodies to deplete CD4^+^ T cells, CD8^+^ T cells, or both CD4^+^ and CD8^+^ T cells, followed by transplantation of the dox-inducible *Gata4* KP lung cell line into pre-depleted syngeneic hosts. Mice received depleting antibodies or an isotype control antibody continuously throughout the experiment to ensure that the target cell type was thoroughly depleted. Flow cytometry-based quantification of CD4^+^ and CD8^+^ T cells in peripheral blood confirmed on-target effects (Supplementary Fig. 9a-d). Depletion of CD8^+^ T cells either alone or in combination with CD4^+^ T cells completely rescued the tumor-suppressive phenotype of *Gata4* (Fig. 4g, Supplementary Fig. 9f). However, CD4 depletion alone could not rescue the effects of *Gata4* overexpression in this setting (Fig. 4g). These results support the initial observations that GATA4 might act in a tumor-suppressive manner and implicate a role for the GATA4-regulated secretome in recruiting the immune system into tumors to limit their initiation and the rate of tumor growth in a cell non-autonomous manner.

**Fig. 5:**
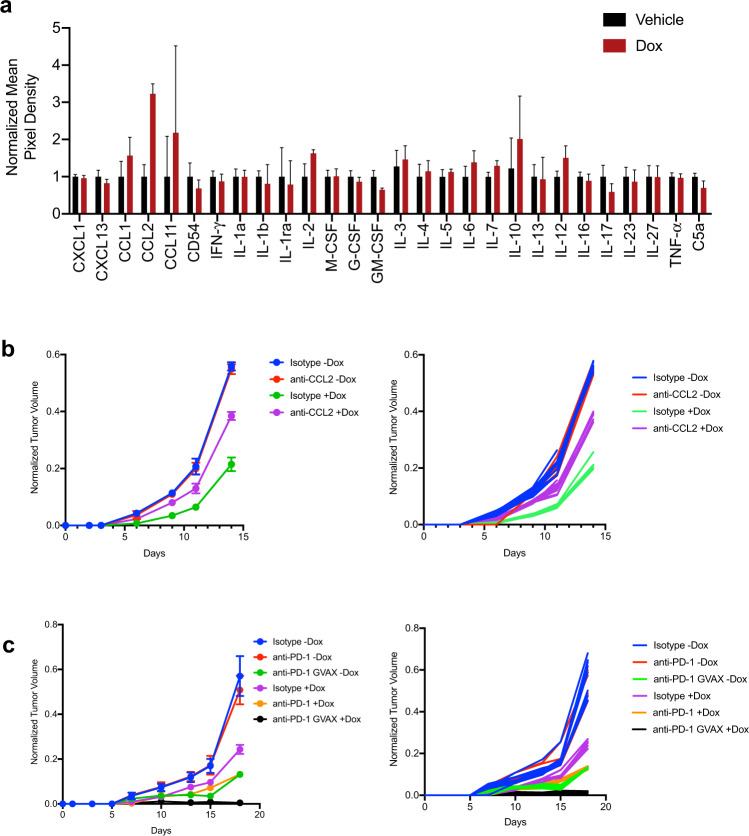
*Gata4* and anti-PD-1 immune checkpoint therapy combine to abrogate tumor growth. **a** Cytokine array of media harvested from KP lung tumor cells with dox-inducible *Gata4* treated with vehicle (black) or dox (red). Data are mean ± SD and *n* = 4 for each group. **b** KP cells with dox-inducible *Gata4* were transplanted into the flanks of immunocompetent syngeneic hosts. Mice were treated with either isotype or anti-CCL2 antibody, with and without dox. Left panel shows aggregated tumor volume data by group with points representing mean ± SD and *n* = 10 per group combined from 2 independent experiments. Right panel shows individual tumor data with each line corresponding with an individual tumor. **c** B16 melanoma cells with dox-inducible *Gata4* were transplanted into the flanks of immunocompetent syngeneic hosts. Mice were treated with either isotype or anti-PD-1 antibody, with and without dox, and also GVAX, with and without dox. Left panel shows aggregated tumor volume data by the group with points representing mean ± SD and *n* = 10 per group combined from 2 independent experiments. Right panel shows individual tumor data with each line corresponding with an individual tumor. For this figure, source data are provided as a Source Data file.

Bulk tumor RNA-seq of somatically edited tumors in the autochthonous KPC model of lung cancer showed numerous changes in the microenvironment of sg*Gata4*-targeted tumors; however, it is not clear which changes can be directly attributed to cells in which *Gata4* was edited. To identify factors secreted by tumor cells directly in response to *Gata4* overexpression, we used cytokine arrays to assay conditioned media from tet-Gata4 and tet-GFP KP tumor cells. We identified CCL2 as a secreted factor produced by KP cells in response to GATA4 pathway activation (Fig. 5a). To determine whether CCL2 was required for *Gata4*-mediated tumor suppression, we transplanted KP tet-*Gata4* tumor cells into immunocompetent syngeneic hosts and divided mice into control or doxycycline groups with either a CCL2-neutralizing antibody or isotype control. CCL2 neutralization partially rescued *Gata4*’s tumor-suppressive phenotype (Fig. 5b).

Immunotherapy via immune checkpoint blockade has emerged as an effective cancer therapy. B16 melanoma cell tumor transplants are responsive to immune checkpoint blockade^45^. To test whether GATA4 pathway activation impacts response to immunotherapy, we transplanted B16 melanoma cells with the tet-Gata4 construct into immunocompetent syngeneic hosts and divided mice into control or doxycycline groups with an anti-PD-1 antibody or isotype control. We also used GVAX, an established cell-based vaccine that primes the host immune system to respond effectively to anti-PD-1 therapy and clear tumors. *Gata4* overexpression combined with anti-PD-1 antibody led to tumor suppression that was not statistically different from GVAX combined with anti-PD-1 antibody. GVAX, *Gata4* overexpression, and anti-PD-1 antibody together efficiently prevented detectible tumor growth (Fig. 5c).

## Discussion

*GATA4* is frequently lost in human cancer and our analyses found that *GATA4* copy number is positively correlated with the number of TILs in multiple human tumor types. While other genes linked to *GATA4* on chromosome 8p may also participate in this phenotype, our in vivo studies in GEMMs and transplant models suggest that *GATA4* perturbation may play a significant role in preventing immune infiltration of tumors. *Gata4* expression serves as a potent tumor-suppressive mechanism in an autochthonous model of *Kras*-driven lung adenocarcinoma, affecting tumor initiation, but not cell proliferation (Fig. 1i). Somatic editing of *Gata4* results in tumors that recruit fewer TILs, and this effect is specific to inactivation of the *Gata4* branch and not to mutation of the senescence regulator *Cdkn2a*, which does not impact TIL recruitment as revealed by RNA-seq data (Supplementary Fig. 3a).

*Gata4* overexpression can mimic its stabilization during senescence induction^17^. When overexpressed in tumor cells transplanted into immune-competent mice, *Gata4* suppressed tumor growth in multiple tumor types, even when only a subset of tumor cells was programmed to express *Gata4*. Importantly, the *Gata4* pathway functioned in a cell non-autonomous, dose-dependent manner to recruit TILs and suppress tumor growth. We did not observe any effect of *Gata4* on the cell cycle in vivo. *Gata4* overexpressing cells were not depleted relative to control tumor cells in the same tumor, revealing that *Gata4* does not function to regulate the cell cycle in a cell-autonomous manner. Importantly, *Gata4* required an intact adaptive immune system, specifically CD8 cytotoxic T cells, in order to suppress tumor growth. This is in contrast to a previously defined role for NK cells in clearance of senescent cells induced by TP53 reactivation in hepatic cancer models^43^ or combinatorial treatment with CDK4/6 and MEK inhibitors^42^. ATM activation by chronic DNA damage is known in certain circumstances to induce NKG2D ligands recognized by NK cells^46^. However, the fact that the *Rag1*^−*/*−^mice or CD8 cell depleted wild-type mice used in our study are proficient in NK cell function yet fully block the effects of *Gata4*-induced tumor suppression suggests that, at least in this genetic context, *Gata4* does not require NK cells to limit tumor growth. We also showed that GATA4’s secretome was necessary for its tumor-suppressive effect by identifying the GATA4-induced secreted factor CCL2 and demonstrate that CCL2 was partially responsible for GATA4’s tumor-suppressive effects in vivo using a CCL2-targeting antibody. Lastly, we showed that *Gata4* overexpression combined with immune checkpoint blockade effectively prevented tumor growth.

These studies provide genetic evidence aiming to specifically disentangle the role of the *Gata4*-controlled secretory program in cancer from that of the known senescence cell cycle regulatory branches. Tumors that have escaped the cell cycle arrest of senescence by mutation of the TP53 and CDKN2A pathways might still be activating a GATA4-dependent secretory response that partially limits tumor growth. However, the degree and frequency of its activation remain to be established. This has important potential ramifications for cancer therapeutics. First, tumors already undergoing a SASP-like response might represent the “hot” tumors that have more TILs and could display higher levels of responsiveness to immune checkpoint therapies. Secondly, a therapeutic strategy that involves increasing this secretory pathway in tumors might synergize with immune checkpoint blockade to more efficiently target tumors. The ability to distinguish GATA4-activated secretory versus GATA4-inactive tumors will be critical to resolving these issues.

During the course of this work, a study was published showing *Gata4* loss was tumorigenic in a *Kras*-driven GEMM with wild-type p53 but found no role for SASP and further suggested that cell proliferation might be responsible for increased tumor burden^30^. It is unclear why no evidence for SASP was found, but as p53 is known to inhibit SASP^19,32^, this difference may be responsible for the discrepancy in results. The presence of TP53 may prevent strong activation of the GATA4 pathway as such strong activation signals might activate TP53 and promote apoptosis or senescence, eliminating those cells from the tumor while selecting for pathways that do not activate SASP in the same manner as the KP mouse model. Indeed, we find that loss of *GATA4* in human tumors occurs largely in the presence of *TP53* mutation or deletion, suggesting a conditional tumor-suppressive role of the GATA4-SASP pathway only in the absence of *TP53*. Which signals and at what strength they are needed to activate the *TP53* versus *GATA4* branches in tumors is not known nor is the composition of the SASP pathway in these contexts. These are important questions for future study.

It is not clear which cells are in a SASP-like state in KP-mutant lung tumors and whether they represent a subset of senescent cells or if all tumor cells are “SASPing”. Given that the vast majority of mouse senescence is p53-dependent, it is likely that the loss of p53 prevents senescence in the classical sense but not parallel pathways regulated by GATA4. However, in the heterotopic tumor models, it is clear that the proliferating non-senescent tumor cells are “SASPing” because their numbers as a proportion of the control tumor cells remain constant throughout tumor progression. Instead, it is a tumor-associated secretory phenotype we can refer to as TASP. How tumors activate TASP and whether it presents the same or a modified SASP pathway remains to be determined. The fact that *GATA4* copy number correlates with TIL infiltration, which is known to be a positive predictive factor for immune checkpoint responsiveness, raises the interesting possibility that SASP/TASP in tumors could be therapeutically advantageous during immune checkpoint therapy.

Together, these studies argue that SASP/TASP enhances the immune scrutiny of cells in a tumor setting by leveraging the adaptive immune system and is likely to do so in the context of other senescence-inducing conditions. Precisely which factors are involved in promoting the influx of the different immune cell classes into tumors and whether these factors act directly to recruit these lymphocytes or work through a cytokine/chemokine cascade that instructs other cells in the stroma to participate in recruitment remains to be determined. Regardless of the precise mechanisms employed, these findings provide a clearer picture of the biological mechanisms underpinning cancer evolution by genetically disentangling the non-cell-autonomous role of SASP/TASP from cell proliferation and suggest potential strategies to counter tumorigenesis.

## Methods

Our research complies with all relevant ethical regulations and has been approved by the Brigham & Women’s Hospital institutional review board and follows their prescribed ethical guidelines.

### TCGA analysis

Genome-wide copy number, RNA-seq, and mutational data were generated by the TCGA Research Network (https://www.cancer.gov/tcga), and downloaded using the Broad GDAC firehose (10.7908/C11G0KM9 (http://gdac.broadinstitute.org/)) for tumor types lung squamous cell carcinoma (LUSC), lung adenocarcinoma (LUAD), breast adenocarcinoma (BRCA), colorectal adenocarcinoma (COAD), skin cutaneous melanoma (SKCM), prostate adenocarcinoma (PRAD), bladder adenocarcinoma (BLCA), uterine corpus endometrial carcinoma (UCEC), liver hepatocellular carcinoma (LIHC), and head and neck squamous cell carcinoma (HNSC). The level three data types used were SNP array-based segmented copy number (minus germline) files for CNA analysis, RSEM normalized files for gene expression analysis, and mutation annotation files (MAF) for exome calling. To determine with the highest confidence tumor samples in which GATA4 is deleted, we first corrected the log_2_ segment mean copy number ratios for tumor purity using previously defined purity estimates based on differential methylation data^47,48^, so as not to confound our results by using purity estimates based on the data types used in downstream analyses (i.e., snp-based allelic data or RNA-seq data). These differential methylation-based purity estimates are highly concordant with ABSOLUTE-based purity estimates^48^. For a log_2_-transformed copy number ratio x, and tumor purity fraction p, we derived a purity-corrected log_2_-transformed copy number ratio c as follows:

$${{{{{\rm{c}}}}}}=\,\log_{2}\left(\frac{{2^{x}}-(1-{{{{{\rm{p}}}}}})}{p}\right)$$

We utilized a threshold for calling GATA4 loss based on purity-corrected copy number ratios corresponding to loss of at least one copy in a triploid tumor background, i.e., loss of 33%.

The fraction of the genome altered by copy number alteration was determined using the same purity-corrected log_2_-transformed copy number ratios used to determine GATA4 CNAs, with cutoffs corresponding to gain or loss of one copy in a triploid background, i.e. gain or loss of 33%. The sum of the lengths of all copy number altered genomic regions was divided by total length of the genome surveyed by snp probes to derive the fraction of the genome altered per tumor. “Low aneuploidy” samples were defined as members of the lowest tercile of the distribution of fractions genome altered. To determine differential gene expression in GATA4-lost versus GATA4-wt/GATA4-gained tumors, we tested for differential distributions of expression levels for every gene measured by RNA-seq in the two groups using edgeR glmFIT and glmRT functions^49,50^. FDR corrections were used to adjust p values for multiple hypothesis testing. FDR-ranked differentially expressed gene lists were used for downstream GSEA analysis.

All arm-level CNAs were determined using the following criteria: gain or loss of at least one copy in a triploid background, with at least 75% of the arm affected. LOF mutations in TP53 were determined based on non-synonymous status in the mutant allele frequency datasets. Hypergeometric tests were used to determine significant overlaps between focal or arm-level CNA status and TP53 mutation/loss status. Gene-based number plots in Supplementary Figs 4 and 5 were made using a modified version of Copy Number Explorer^51^.

Cohort normalization (CNorm) of CNA landscapes was performed using copy number calls for each tumor based on the above criteria. We first calculated the population frequencies of all CNAs in GATA4-deleted and GATA-wt sample groups. We then categorized CNAs as either enriched in GATA4-deleted tumors or enriched in GATA4-wt tumors, if the difference in frequencies of the CNA in the two populations was > 5%. We then assigned a score to each sample in each group equivalent to the number of group-specific over-represented CNAs present in the sample. We then removed several samples with the highest overrepresentation scores at random and recalculated all CNA frequencies in each group. We repeated this process iteratively until all CNAs were at near-equivalent frequencies in each population. If perfect normalization could not be achieved due to very strong co-associations, we achieved the highest level of CNA frequency concordance possible while removing no more than 50% of samples in either group.

### GSEA

For the TCGA analysis, GSEA analysis using the GSEA PreRanked weighted mode^37^ using custom, published genesets^52^ specific to immune cell subtypes was applied to the ranked list of genes described above. The FDR q-value for the degree of positive correlation between each immune cell subtype-specific geneset and GATA4 copy number is shown in Fig. 1d. For analysis of mouse tumors, GSEA of analysis using the GSEA PreRanked weighted mode using the Reactome, Hallmark, and Biocarta databases was applied to ranked gene lists considering all of the available gene signatures from the databases. The top-ranked pathways, or a subset of top-ranked pathways, are displayed in Fig. 3a. The FDR q-values for each displayed geneset are less than 0.05.

### Lentiviral vectors and sgRNA cloning

The pUSEC lentiviral vector and cloning strategies were previously described^36^. For sgRNA cloning, all vectors were digested with BsmBI and ligated with BsmBI-compatible annealed oligos for sgRNAs and cloned as previously described (Supplementary Sequences). pInducer21 and pInducer30 both had the murine *Gata4* gene and the control GFP gene cloned into them. In each case, to control for the immunogenicity of the control GFP gene, GFP was used as the selection marker for infection so that it was present in all cells.

### Lentiviral production

Lentiviruses were produced by co-transfection of HEK 293 cells with lentiviral backbone constructs and packaging vectors (psPAX2 and pMD2.G; Addgene #12260 and #12259) using TransIT-LT1 (Mirus Bio #MR 2306). The supernatant was collected 48 and 72 h post-transfection, concentrated by ultracentrifugation at 9000 × *g* for 120 min and resuspended in an appropriate volume of OptiMEM (Gibco #31985-062).

### Cell culture

All cells were cultured at physiologic O_2_ levels using low-oxygen incubators. All cells were cultured using DMEM (Gibco #21013024) with 10% FBS (GE Life Sciences #SH30071.03).

### Mice

All animal studies described in this study were approved by the Brigham & Women’s Hospital Institutional Animal Care and Use Committee or the MIT Institutional Animal Care and Use Committee ethical guidelines. *Kras*^*LSL-G12D/+*^; *p53*^*fl/fl*^; *Rosa26*^*LSL-Cas9*^ (KPC) mice have already been described^36^. For all animal studies, >3 animals were used for each experimental cohort per specified genotype. KPC mice were maintained on a mixed C57BL/6:SV129 genetic background. C57BL/6J mice and CD57BL/6J; *Rag1*^−/−^ mice were acquired from Jackson Labs and were of both sexes. All mice were ordered at ~12 weeks of age. Total burden and grading analyses were conducted on > 3 mice per genotype. Animals lacking detectable tumors by histopathology were excluded from the analysis to ensure all animals were properly infected with pUSEC lentiviruses. Animals with the appropriate genotypes between the ages of 6–10 weeks were randomly selected to begin tumor initiation studies with pUSEC lentiviruses. Mice were infected intratracheally with 25,000 TU’s of lentiviruses as described^34^. Total lung area occupied by tumor was measured on hematoxylin and eosin (H&E) stained slides using NIS-elements software. Histological quantification of mouse lung tumor burden by grade was performed by an automated deep neural network developed by Aiforia Technologies in collaboration with the Jacks lab, and in consultation with veterinarian pathologist Dr. Roderick Bronson. The algorithm performed consistently and with high correlation with human graders across multiple validation datasets independent of the training dataset. The maximal tumor size permitted by the institutional review board is 1 cubic centimeter and we have complied with this policy.

### Immunohistochemistry

Mice were euthanized by carbon dioxide asphyxiation. Lungs were perfused through the trachea with 4% paraformaldehyde (PFA), fixed overnight, transferred to 70% ethanol and subsequently embedded in paraffin. Sections were cut at a thickness of 4 um and stained with H&E for pathological examination. Chromogenic immunohistochemistry (IHC) was performed on a Ventana Medical Systems Discovery XT instrument with online deparaffinization using Ventana’s reagents and detection kits and antigen retrieved in Ventana Cell Conditioner 1 or 2. The following antibodies were used for IHC: anti-phospho-Histone H3 (pHH3) (Ser10; Cell Signaling, 9701, 1:200), CD3 (Abcam, ab16669, 1:200), CD8 (Abcam, ab217344, 1:200). Horseradish peroxidase (HRP) detection was used for all and was antigen retrieved in Ventana Cell Conditioner 1 (Tris-Borate-EDTA). Pictures were obtained using a digital whole slide scanner Leica SCN400F and Slidepath software version 4.0.8.

### Bioinformatic analysis of CRISPR-targeted loci

For PCR amplicons (sequenced at the MGH sequencing facility), 150–300 bp paired-end reads were used in downstream analyses. The reference sequence of the target locus was supplemented with 10 bp genomic flanks and was indexed using an enhanced suffix array^53^. Read ends were anchored in the reference sequence using 10 bp terminal segments for a suffix array index lookup to search for exact matches. A sliding window of unit step size and a maximal soft-clip limit of 10 bp was used to search for possible anchors at either end of each read. For each read, optimal Smith-Waterman dynamic programming alignment^54^ was performed between the reduced state space of the read sequence and the corresponding reference sequence spanning the maximally distanced anchor locations. Scoring parameters were selected to allow for sensitive detection of short and long insertions and deletions while allowing for up to four mismatches and the highest-scoring alignment was selected. Read pairs with both reads aligned in the proper orientation were processed to summarize the number of wild-type reads and the location and size of each insertion and deletion event. Overlapping reads within pairs were both required to support the event if they overlapped across the event location. Additionally, mutation events and wild-type reads were summarized within the extents of the sgRNA sequence and PAM site by considering read alignments that had a minimum of 20 bp overlap with this region. Mutation calls were translated to genomic coordinates and subsequently annotated using Annovar^55^. The alignment and post-processing code was implemented in C + + along with library functions from SeqAn^56^ and SSW and utility functions in Perl and R (www.R-project.org). Mutation calls were subjected to manual review using the Integrated Genomics Viewer^57^.

### Transcriptional profiling

Tumor tissues were frozen in RNAlater solution (Thermo #PA5-39542) and RNA was extracted using the RNeasy plus mini kit (Qiagen #74134). cDNA libraries were built using NEB Next Ultra RNA Library Prep Kit for Illumina (NEB #E7530S). Samples were multiplexed using NEB Next Multiplex Oligos for Illumina (NEB #E7710L). Samples were sequenced on an Illumina NexSeq 500 and reads were aligned to the mouse genome (GRCm38) using HiSat2^58^. Mapped reads were counted using the featureCounts function from the Subread^59^ package. Differential expression between groups was calculated using edgeR^50^.

### Antibody-mediated depletion

CD4-depleting antibody (#BP0003-1), CD8-depleting antibody (#BP0061), and the isotype control antibody (#BP0090) were acquired from BioXCell. Antibodies were diluted using dilution buffer (BioXCell #IP0070) to 100 ug/100 uL. 200ug of the relevant antibody mixture (100ug CD4-depleting antibody plus 100 ug isotype control antibody, 100ug CD8-depleting antibody plus 100ug isotype control antibody, 200 ug isotype control antibody, or 100 ug CD4-depleting antibody plus 100 ug CD8-depleting antibody) were given to each mouse 1 day before tumor cell transplant and every 3 days subsequently. Circulating lymphocytes were profiled by collecting peripheral blood and performing flow cytometric analysis.

### Antibody-mediated neutralization of CCL2

CCL2-neutralizing antibody (#BE0185) and its isotype control were acquired from BioXCell. Antibodies were diluted using dilution buffer (BioXCell #IP0070) to 100 ug/100 uL.

### Immune checkpoint blockade with anti-PD-1 antibody

Anti-PD-1 antibody (#BP0273) and its isotype control were acquired from BioXCell.

### Reporting summary

Further information on research design is available in the Nature Research Reporting Summary linked to this article.

## Supplementary information

Supplementary Information

Description of Additional Supplementary Files

Supplementary Data 1

Supplementary Data 2

Supplementary Data 3

Supplementary Data 4

Supplementary Data 5

Reporting Summary

## Data Availability

The RNA-Seq data are deposited in the Sequences Read Archive (SRA) with the project accession number PRJNA778768. The remaining data are available within the Article, Supplementary Information or Source Data file. Source data are provided with this paper.

## Source data

Source Data

## Code availability

The source code used to analyze data are provided at the following publicly available repository: https://github.com/emmavwatson/CNorm.
