# Supplementary material for: A GATA4-regulated secretory program suppresses tumors through recruitment of cytotoxic CD8 T cells
Source: Nat Commun. 2022 Jan 11;13:256. doi: 10.1038/s41467-021-27731-5 (PMC8752777; doi:10.1038/s41467-021-27731-5)
Supplement: Supplementary file 1 — Supplementary Information [file 41467_2021_27731_MOESM1_ESM.pdf]

Supplementary Information

A GATA4-Regulated Secretory Program Suppresses Tumors through recruitment of Cytotoxic CD8 T cells

Rupesh S. Patel[1,2,3], Rodrigo Romero[4,5], Emma V. Watson[1,2,3], Anthony C. Liang[1,2,3], Megan Burger[4], Peter M.K. Westcott[4], Kim L. Mercer[4], Roderick T. Bronson[6], Eric C. Wooten[1,2,3], Arjun Bhutkar[4], Tyler Jacks[3,4,5], Stephen J. Elledge[1,2,3*]

[1] Division of Genetics, Department of Medicine, Brigham and Women's Hospital, Boston, MA, USA

[2] Department of Genetics, Harvard Medical School, Boston, MA, USA

[3] Howard Hughes Medical Institute, Chevy Chase, Maryland, USA

[4] David H. Koch Institute for Integrative Cancer Research, Massachusetts Institute of Technology, Cambridge, MA, USA

[5] Department of Biology, Massachusetts Institute of Technology, Cambridge, MA, USA

[6] Harvard Medical School, Boston, MA, USA

*Corresponding author selledge@genetics.med.harvard.edu

Supplementary Figure 1 Characterization of somatically edited tumors

(A) Quantification of the distribution of tumor grades across KP mice 16 weeks P.I. Tumor grade is established by analyzing H&E stains of serial lung tissue sections using an algorithm developed by the Jacks laboratory. The percent of tumor area in each grade is defined as the percent of total lung area that contains tumor tissues of that grade. n=10 for sgCtrl and sgGata4 and n=4 for sgCdkn2a.

(B) Quantification of the distribution of tumor grades across KP mice 8 weeks P.I. Tumor grade is established by analyzing H&E stains of serial lung tissue sections using an algorithm developed by the Jacks laboratory. The percent of tumor area in each grade is defined as the percent of total lung area that contains tumor tissues of that grade. n=3 mice from sgCtrl, n=4 mice from sgGata4.1, and n=4 mice from sgGata4.12.

(C) CRISPR-seq of *Gata4* locus in sg*Gata4* and sg*Ctrl* targeted mice. Sequencing is performed on 1kB amplicons centered on the predicted sgRNA cut site. The mutational burden is not corrected for tumor purity and therefore is an underestimate of true editing efficiency. Each bar represents the mutational distribution of reads within a single tumor. n=11 tumors for sgGata4 and n=8 tumors for sgCtrl.

(D) CRISPR-seq of *Cdkn2a* locus in sg*Cdkn2a* and sg*Ctrl* targeted mice. Sequencing is performed on 1kB amplicons centered on the predicted sgRNA cut site. The mutational burden is not corrected for tumor purity and therefore is an underestimate of true editing efficiency. Each bar represents the mutational distribution of reads within a single tumor. n=10 tumors for sgCdkn2a and n=8 tumors for sgCtrl.

(E) Representative images of IHC staining for pHH3 (left) and quantification of IHC for pHH3 staining (right) on serial sections of lung tissue at 8 weeks P.I. Statistical test is Mann-Whitney test. Data are mean ± SEM and n=4 mice and 48 images for sgCtrl and n=6 mice and 325 images for sgGata4. Experiment performed once.

Fig. S1:

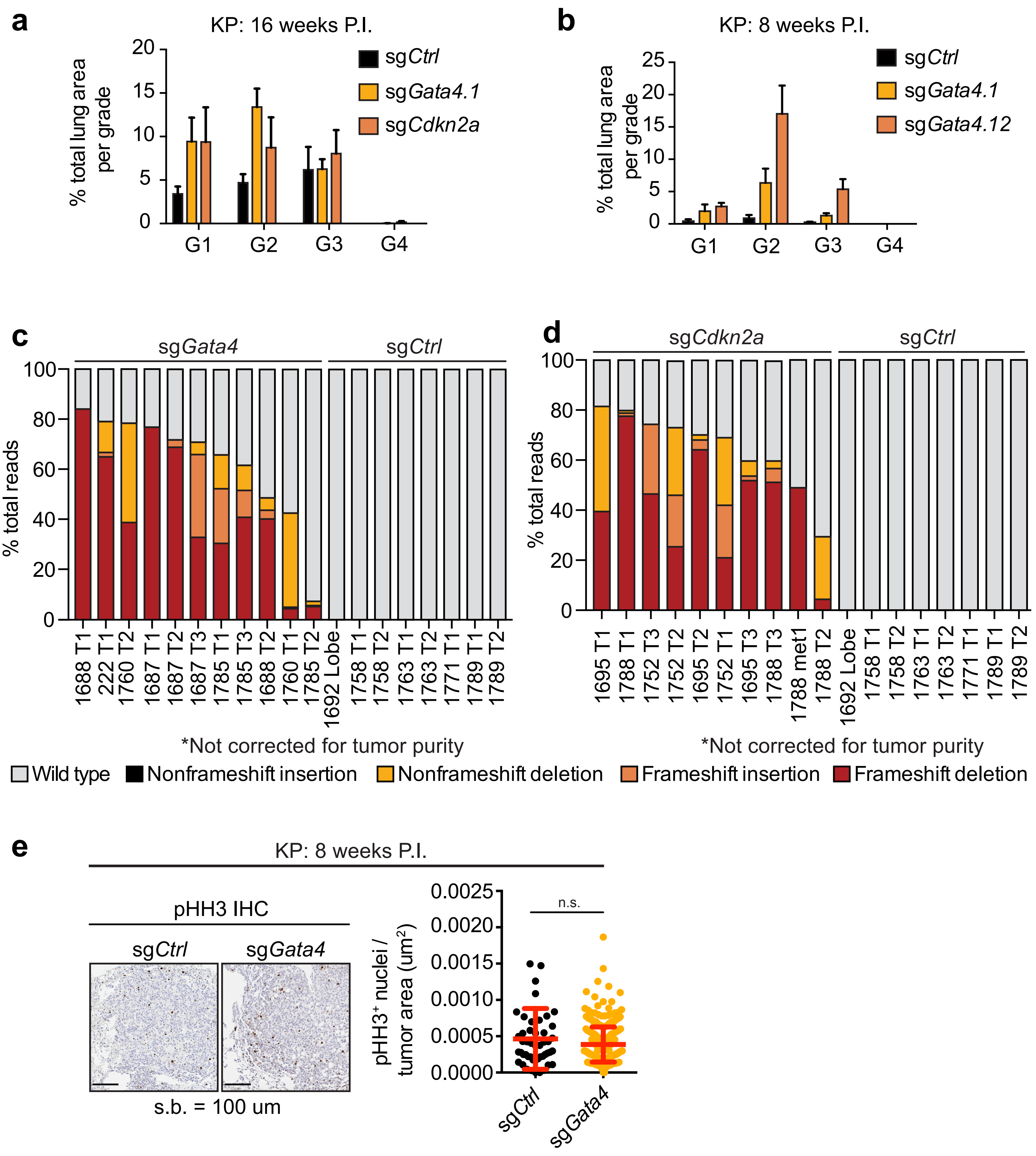

a
KP: 16 weeks P.I.
% total lung area per grade
sgCtrl
sgGata4.1
sgCdkn2a
G1
G2
G3
G4
b
KP: 8 weeks P.I.
% total lung area per grade
sgCtrl
sgGata4.1
sgGata4.12
c
sgGata4
sgCtrl
% total reads
1688 T1
222 T1
1760 T2
1687 T1
1687 T2
1687 T3
1785 T1
1785 T3
1688 T2
1760 T1
1785 T2
1692 Lobe
1758 T1
1758 T2
1763 T1
1763 T2
1771 T1
1789 T1
1789 T2
*Not corrected for tumor purity
d
sgCdkn2a
sgCtrl
% total reads
1695 T1
1788 T1
1752 T3
1752 T2
1695 T2
1752 T1
1695 T3
1788 T3
1788 met1
1788 T2
1692 Lobe
1758 T1
1758 T2
1763 T1
1763 T2
1771 T1
1789 T1
1789 T2
*Not corrected for tumor purity
Wild type
Nonframeshift insertion
Nonframeshift deletion
Frameshift insertion
Frameshift deletion
e
KP: 8 weeks P.I.
pHH3 IHC
sgCtrl
sgGata4
s.b. = 100 um
pHH3+ nuclei / tumor area (um2)
n.s.
sgCtrl
sgGata4

Supplementary Figure 2 Analysis of infiltrating immune cells in Gata4-targetd tumors and controls

(A) Representative images of IHC for CD3 (left) and CD8 (right) at 16 weeks P.I.

(B) Sets of genes whose expression correlates with the abundance of different subsets of TILs (see Supplementary Table 1) are analyzed for their expression in sg*Gata4*-targeted tumors (n=12) versus sg*Ctrl*-targeted tumors (n=6) collected 16-weeks P.I. from the autochthonous KP lung cancer model. Utilized gene sets were derived from single-cell RNAseq data from the Christophe Benoist lab at Harvard Medical School or the Arlene Sharpe lab at Harvard Medical School (Supplementary Table 1). FDR q-values which show the enrichment of the gene sets are derived from GSEA.

Fig. S2:

a

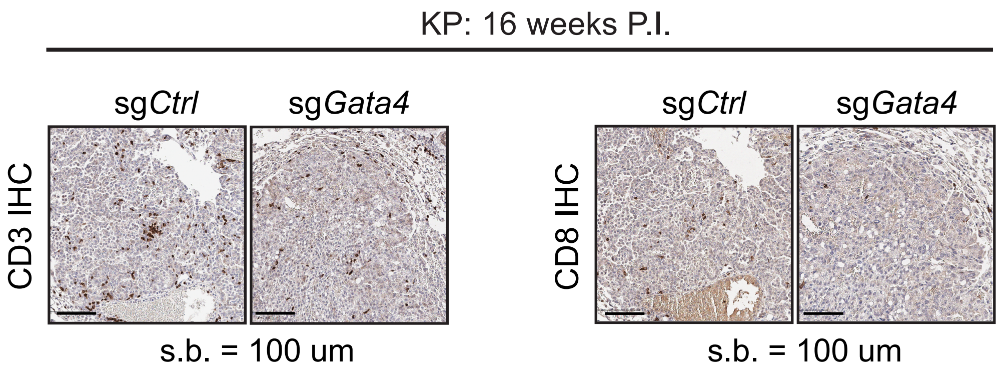
KP: 16 weeks P.I.
sgCtrl
sgGata4
CD3 IHC
s.b. = 100 um
sgCtrl
sgGata4
CD8 IHC
s.b. = 100 um

b

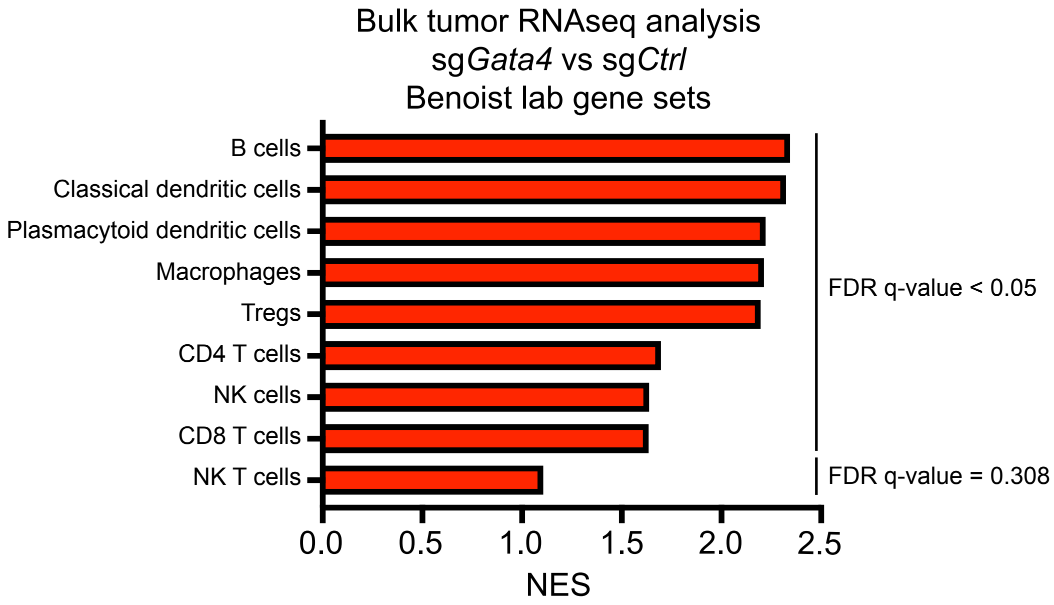
Bulk tumor RNAseq analysis
sgGata4 vs sgCtrl
Benoist lab gene sets
B cells
Classical dendritic cells
Plasmacytoid dendritic cells
Macrophages
Tregs
CD4 T cells
NK cells
CD8 T cells
NK T cells
FDR q-value < 0.05
FDR q-value = 0.308
0.0
0.5
1.0
1.5
2.0
2.5
NES

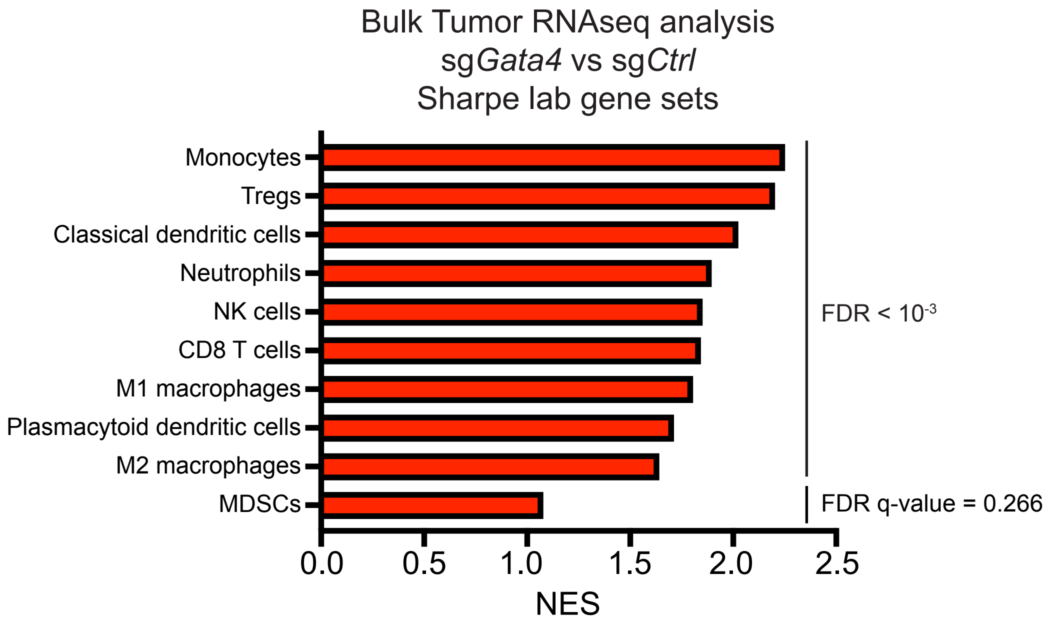
Bulk Tumor RNAseq analysis
sgGata4 vs sgCtrl
Sharpe lab gene sets
Monocytes
Tregs
Classical dendritic cells
Neutrophils
NK cells
CD8 T cells
M1 macrophages
Plasmacytoid dendritic cells
M2 macrophages
MDSCs
FDR < 10-3
FDR q-value = 0.266
0.0
0.5
1.0
1.5
2.0
2.5
NES

Supplementary Figure 3 Cdkn2a-targeted tumors are distinct from Gata4-targeted tumors

(A) Sets of genes specific for different subtypes of TILs (see Supplementary Table 1) are analyzed for their expression in sg*Cdkn2a*-targeted tumors (n=8) versus sg*Ctrl*-targeted tumors (n=6) collected 16-weeks P.I. from the autochthonous KP lung cancer model. Representative genes characterizing each TIL subtype are shown with a heat map representing the corresponding FDR q-value derived from the RNAseq analysis of tumors.

(B) Differential expression of genes involved in antigen presentation from the RNAseq of sg*Gata4*-targeted tumors and sg*Ctrl*-targeted tumors. P-values are from Mann-Whitney tests.

Fig. S3:

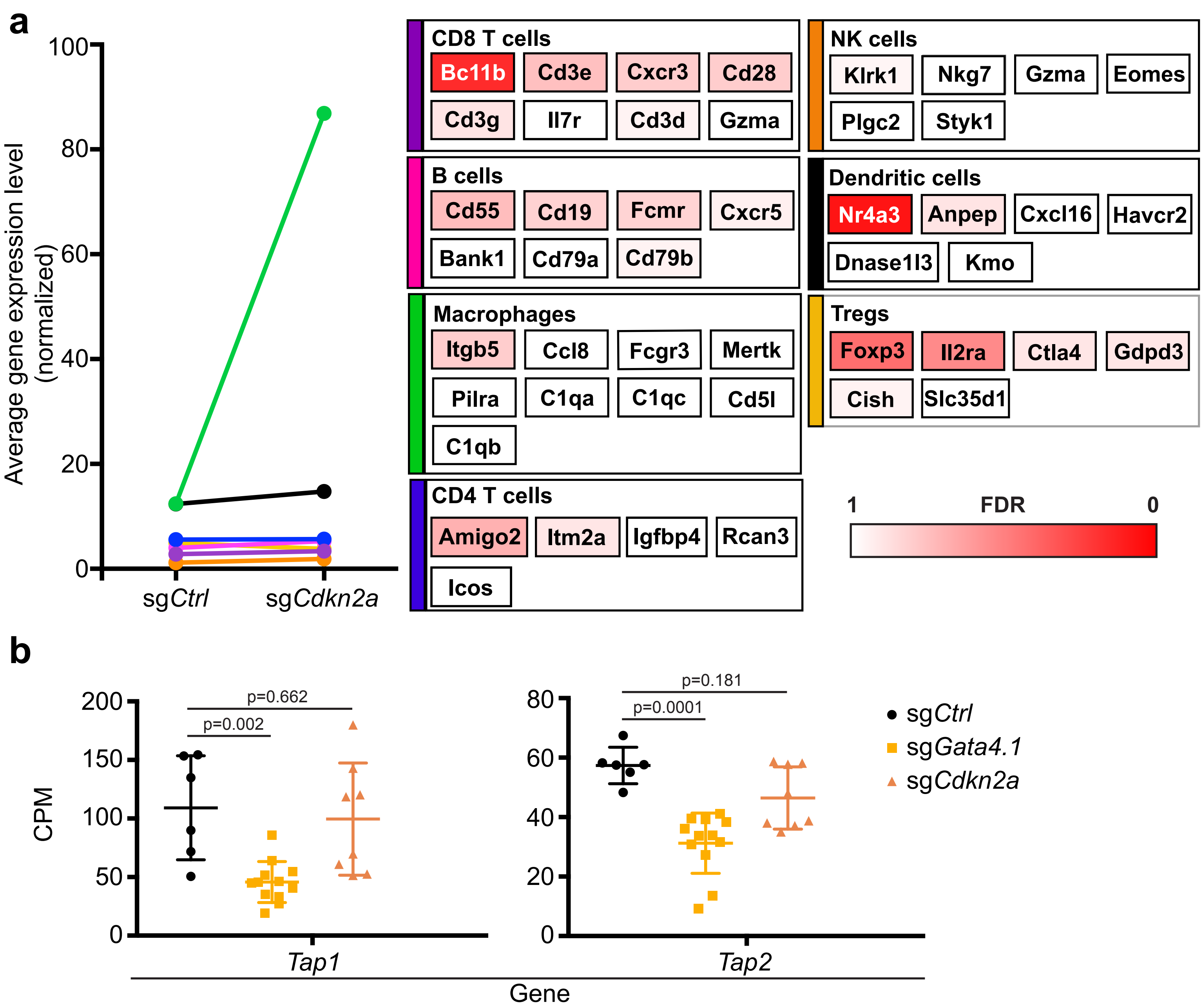
a
Average gene expression level (normalized)
100
80
60
40
20
0
sgCtrl
sgCdkn2a
CD8 T cells
Bc11b Cd3e Cxcr3 Cd28
Cd3g Il7r Cd3d Gzma
NK cells
Klrk1 Nkg7 Gzma Eomes
Plgc2 Styk1
B cells
Cd55 Cd19 Fcmr Cxcr5
Bank1 Cd79a Cd79b
Dendritic cells
Nr4a3 Anpep Cxcl16 Havcr2
Dnase1l3 Kmo
Macrophages
Itgb5 Ccl8 Fcgr3 Mertk
Pilra C1qa C1qc Cd5l
C1qb
Tregs
Foxp3 Il2ra Ctla4 Gdpd3
Cish Slc35d1
CD4 T cells
Amigo2 Itm2a Igfbp4 Rcan3
Icos
1 FDR 0
b
CPM
200
150
100
50
0
p=0.662
p=0.002
Tap1
80
60
40
20
0
p=0.181
p=0.0001
Tap2
Gene
sgCtrl
sgGata4.1
sgCdkn2a

Supplementary Figure 4 Somatic copy number alterations across various cancer types

(A) Summary of TCGA data showing the most common chromosomal deletions (blue) and amplifications (red) in human tumor samples of various tumor subtypes. Chromosome arm 8p contains the human *GATA4* gene. Chromosome 8 is emphasized. Abbreviations are breast adenocarcinoma (BRCA, n=722), lung adenocarcinoma (LUAD, n=448), melanoma (SKCM, n=104), colorectal adenocarcinoma (COAD, n=283), bladder adenocarcinoma (BLCA, n=408), prostate adenocarcinoma (PRAD, n=420), liver hepatocellular carcinoma (LIHC, n=372), lung squamous cell carcinoma (LUSC, n=356), uterine corpus endometrial carcinoma (UCEC, n=426), and head and neck squamous cell carcinoma (HNSC, n=522).

(B) Summary of copy number changes tiling across chromosome 17 in human LUAD samples in TCGA. *TP53* is located on 17p (marked with a vertical black line) which is often lost in human LUAD.

(C) Schematic from cBioPortal showing that *GATA4* is lost in 51% of human lung adenocarcinoma samples in TCGA.

Fig. S4:

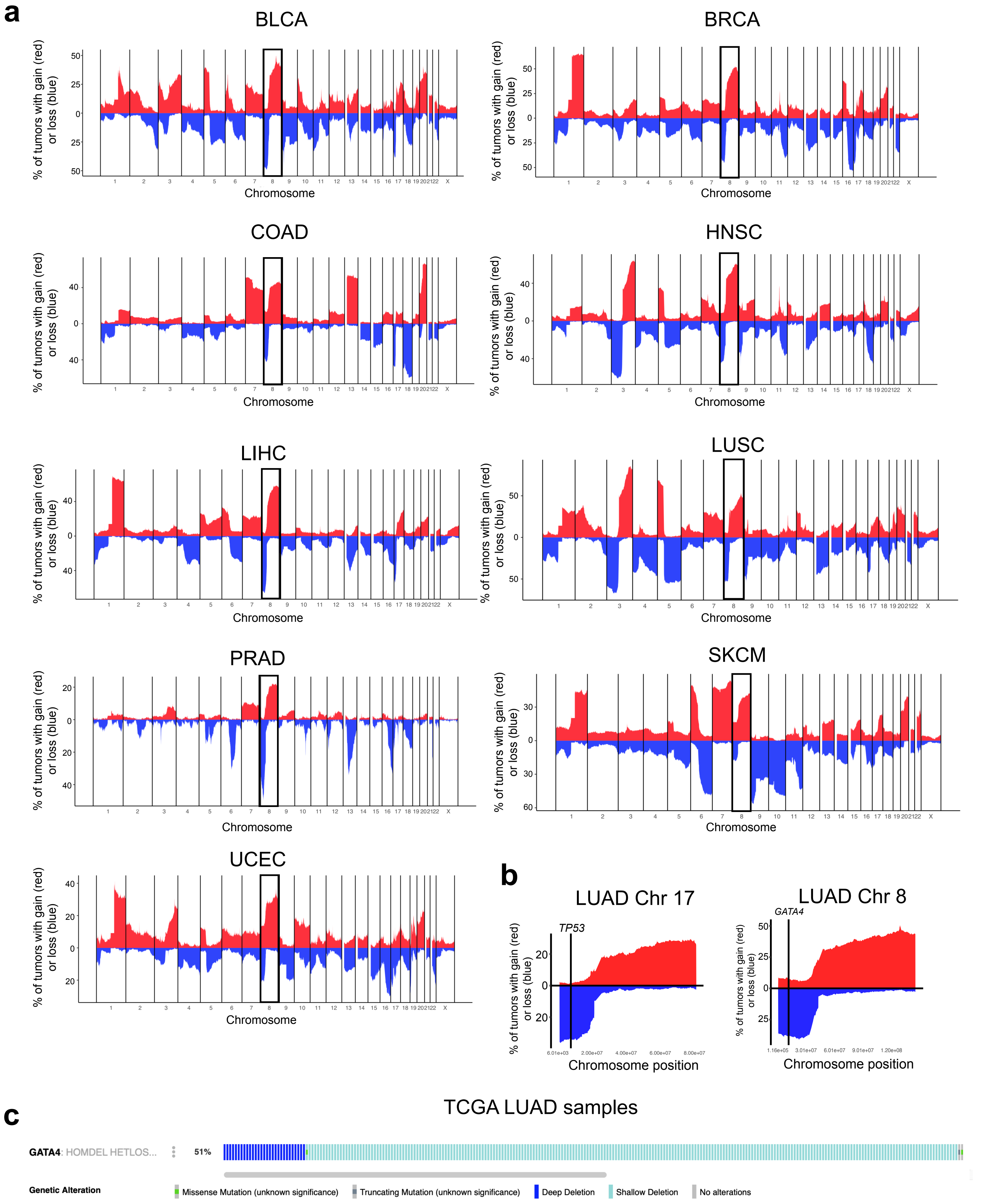
a
BLCA
BRCA
COAD
HNSC
LIHC
LUSC
PRAD
SKCM
UCEC
% of tumors with gain (red) or loss (blue)
Chromosome
b
LUAD Chr 17
TP53
LUAD Chr 8
GATA4
Chromosome position
c
TCGA LUAD samples
GATA4: HOMDEL HETLOS...
51%
Genetic Alteration
Missense Mutation (unknown significance)
Truncating Mutation (unknown significance)
Deep Deletion
Shallow Deletion
No alterations

Supplementary Figure 5 Co-occurrence of *GATA4* loss with *TP53* mutation or 17p deletion

(A) Heatmap displaying the correlation between loss of various chromosome arms and loss of *TP53* (mutation or deletion) in BRCA and LUAD. Most frequent arm losses are shown. P-values are calculated using hypergeometric tests. The color of the cell is determined by the p-value reflecting how often the two events co-occur relative to how often they would be expected to co-occur based on chance alone.

(B) Schematic of human chromosome 8 showing the *GATA4* (deleted in human cancer) and *MYC* (amplified in human cancer) genes.

(C) Summary of copy number changes tiling across chromosome 8 in TCGA tumor samples across various cancer subtypes. *GATA4* is marked with a vertical black line on chromosome arm 8p. Deletions (blue) and amplifications (red) are shown.

Fig. S5:

a

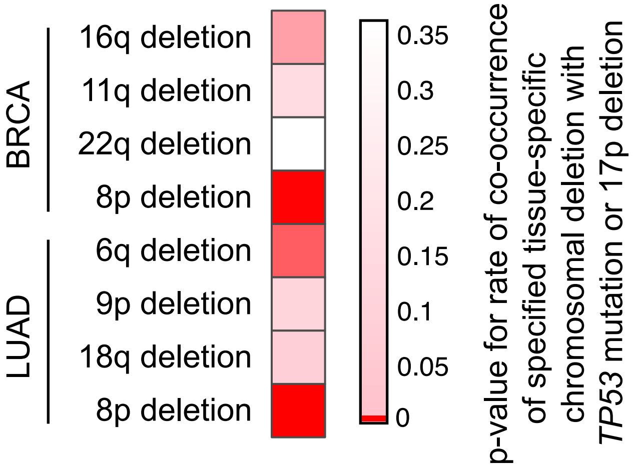
BRCA
LUAD
16q deletion
11q deletion
22q deletion
8p deletion
6q deletion
9p deletion
18q deletion
8p deletion
0.35
0.3
0.25
0.2
0.15
0.1
0.05
0
p-value for rate of co-occurrence of specified tissue-specific chromosomal deletion with *TP53* mutation or 17p deletion

b

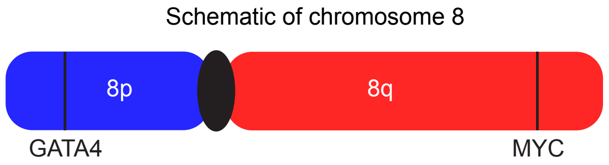
Schematic of chromosome 8
8p
8q
GATA4
MYC

c

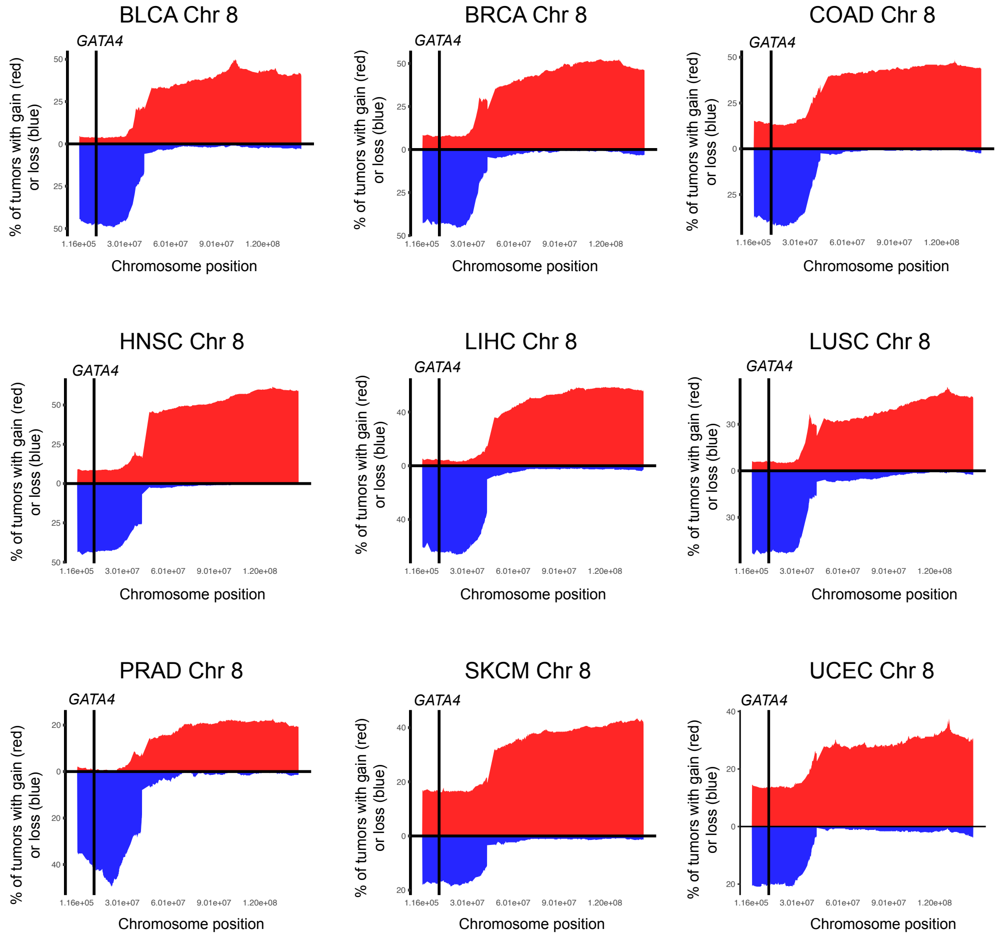
BLCA Chr 8
BRCA Chr 8
COAD Chr 8
HNSC Chr 8
LIHC Chr 8
LUSC Chr 8
PRAD Chr 8
SKCM Chr 8
UCEC Chr 8
GATA4
% of tumors with gain (red) or loss (blue)
Chromosome position
1.16e+05
3.01e+07
6.01e+07
9.01e+07
1.20e+08

Supplementary Figure 6 GSEA analysis of expression of CD8 T cell specific genes in tumors without reduced GATA4 copy number versus tumors with reduced GATA4 copy number

(A) Gene set enrichment analysis showing the distribution of the correlation between CD8 T cell-specific genes and GATA4 copy number in lung adenocarcinoma samples from TCGA. Genes located on the left side of the plot are positively correlated with GATA4 copy number, meaning that their transcripts in tumors fall as GATA4 copy number falls. The log2FC of the expression of genes in tumors without *GATA4* loss versus tumors with *GATA*4 loss is shown. n=120 *GATA4* loss, n=319 no *GATA4* loss tumors.
(B) Gene set enrichment analysis showing the distribution of the correlation between CD8 T cell-specific genes and *GATA4* copy number in human breast adenocarcinoma samples from TCGA. n=256 *GATA4* loss, n=460 no *GATA4* loss tumors.
(C) Gene set enrichment analysis showing the distribution of the correlation between CD8 T cell-specific genes and *GATA4* copy number in human melanoma samples from TCGA. n=20 *GATA4* loss, n=82 no *GATA4* loss tumors.
(D) High levels of aneuploidy correlate with reduced immune infiltration[43] but it is likely that alterations of individual loci also impact how tumors interface with the immune system. To deconvolute whether the association of *GATA4* copy number and immune infiltrate is a byproduct of co-association with general aneuploidy, we limited our analysis to a subset of breast cancer TCGA samples with low levels of aneuploidy (< 20% of genome affected). The association of CD8 T cell-specific genes and GATA4 copy number in human breast adenocarcinoma samples among the low-aneuploid tumors is shown; *GATA4* copy number is still positively correlated with the abundance of TIL-associated transcripts in tumors with low overall levels of aneuploidy.

Fig. S6:

a

CD8 T cells
Lung adenocarcinoma
NES = 1.75
FDR q-value = 0.003

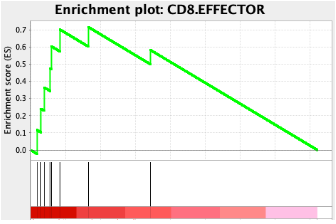

High ⟷ Low

Relative abundance of transcripts in tumors with *GATA4* versus tumors with *GATA4* deletion

Gene	Log2FC
**CD3D**	**0.30**
**KLRG1**	**0.37**
**CD3E**	**0.36**
**CD28**	**0.29**
**CD3G**	**0.23**
**CXCR3**	**0.07**
**IL7R**	**0.37**
**BCL11B**	**0.45**

b

CD8 T cells
Breast adenocarcinoma
NES = 2.06
FDR q-value = 0

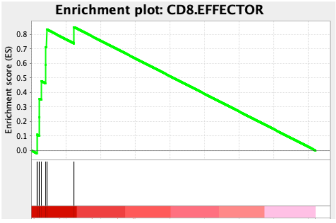

High ⟷ Low

Relative abundance of transcripts in tumors with *GATA4* versus tumors with *GATA4* deletion

Gene	Log2FC
**CD3D**	**0.69**
**KLRG1**	**0.47**
**CD3E**	**0.69**
**CD28**	**0.48**
**CD3G**	**0.65**
**CXCR3**	**0.54**
**IL7R**	**0.67**
**BCL11B**	**0.33**

c

CD8 T cells
Melanoma
NES = 2.08
FDR q-value = 0

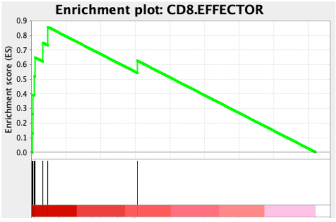

High ⟷ Low

Relative abundance of transcripts in tumors with *GATA4* versus tumors with *GATA4* deletion

Gene	Log2FC
**CD3D**	**1.30**
**KLRG1**	**0.16**
**CD3E**	**1.32**
**CD28**	**0.70**
**CD3G**	**1.14**
**CXCR3**	**1.21**
**IL7R**	**0.95**
**BCL11B**	**1.55**

d

CD8 T cells
Breast adenocarcinoma
Controlled for aneupoloidy
NES = 1.63
FDR q-value = 0.014

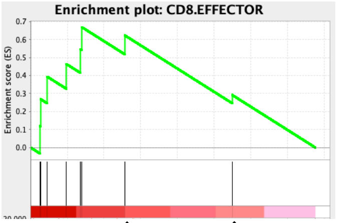

High ⟷ Low

Relative abundance of transcripts in tumors with *GATA4* versus tumors with *GATA4* deletion

Gene	Log2FC
**CD3D**	**0.74**
**KLRG1**	**0.45**
**CD3E**	**0.66**
**CD28**	**0.50**
**CD3G**	**0.49**
**CXCR3**	**0.44**
**IL7R**	**0.34**
**BCL11B**	**-0.12**

Supplementary Figure 7 Immune infiltration phenotype of 8p loss is not due to 8q copy number gain

(A) Some CNAs in human tumors covary, such as +8q with -8p, so to test whether +8q may be driving the immune evasion associated with loss of *GATA4*/8p we calculated the immune infiltrate in *GATA4*/8p loss tumors with or without +8q. Graph showing the NES from GSEA analysis for sets of genes specific to subsets of TILs in BRCA tumors with 8p deletion, including tumors with 8p deletion and 8q gain, compared to tumors without 8p deletion (black), alongside NES for TIL subsets in BRCA tumors with 8p deletion without 8q gain compared to tumors without 8p deletion and without 8q gain.
(B) To attempt to equalize all co-varying CNAs between comparison groups, we developed CNorm to generate custom CNA-normalized tumor cohorts for analyzing impacts of individual mutations/CNAs while minimizing the broader effects of genomic covariates on transcriptomes. Incidences (% cohort) of arm-level copy number alterations in the TGCA lung adenocarcinoma dataset, in tumors with (red) or without (blue) *GATA4* copy number loss. Top: incidences in total cohort (n=120 *GATA4* loss, n=319 no *GATA4* loss). Bottom: incidences in the sub-cohort generated by CNorm (n=63 *GATA4* loss, n=277 no *GATA4* loss).

Fig. S7:

a

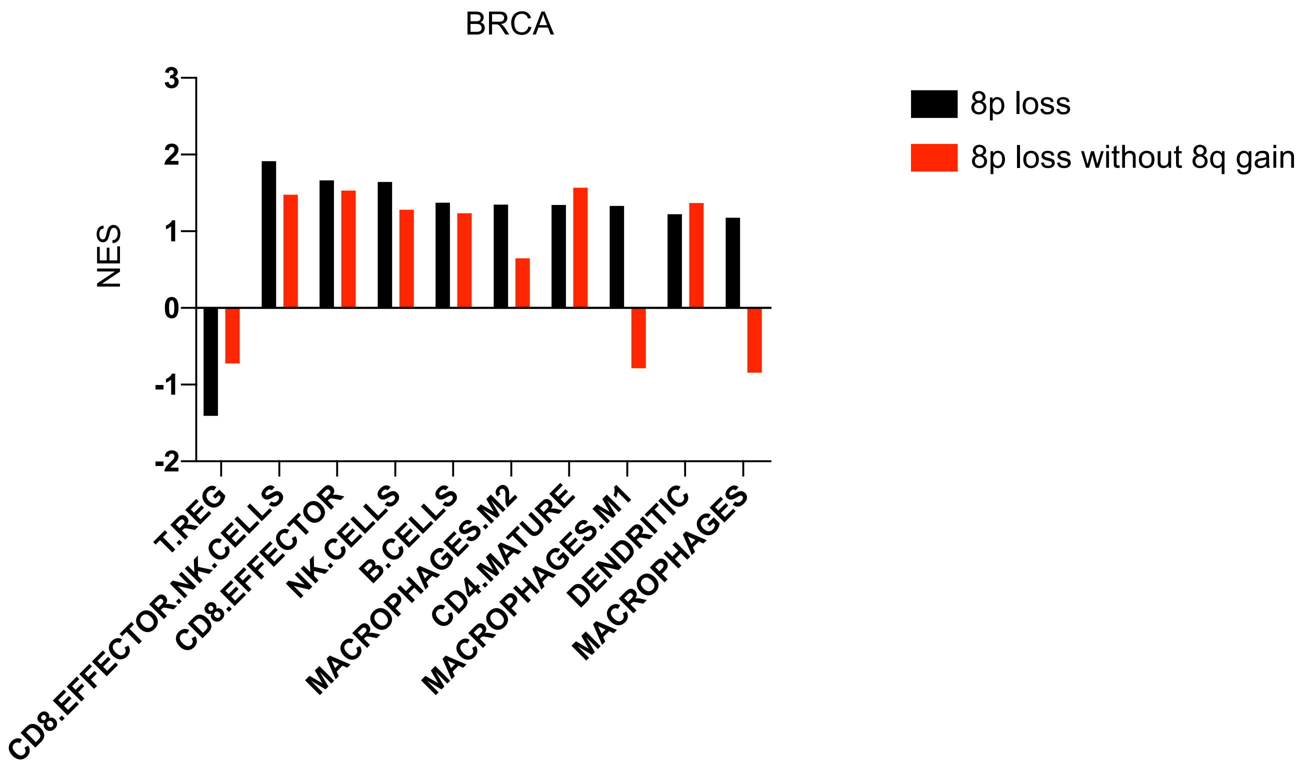
BRCA
8p loss
8p loss without 8q gain
NES
3
2
1
0
-1
-2
T.REG
CD8.EFFECTOR.NK.CELLS
CD8.EFFECTOR
NK.CELLS
B.CELLS
MACROPHAGES.M2
CD4.MATURE
MACROPHAGES.M1
DENDRITIC
MACROPHAGES

b

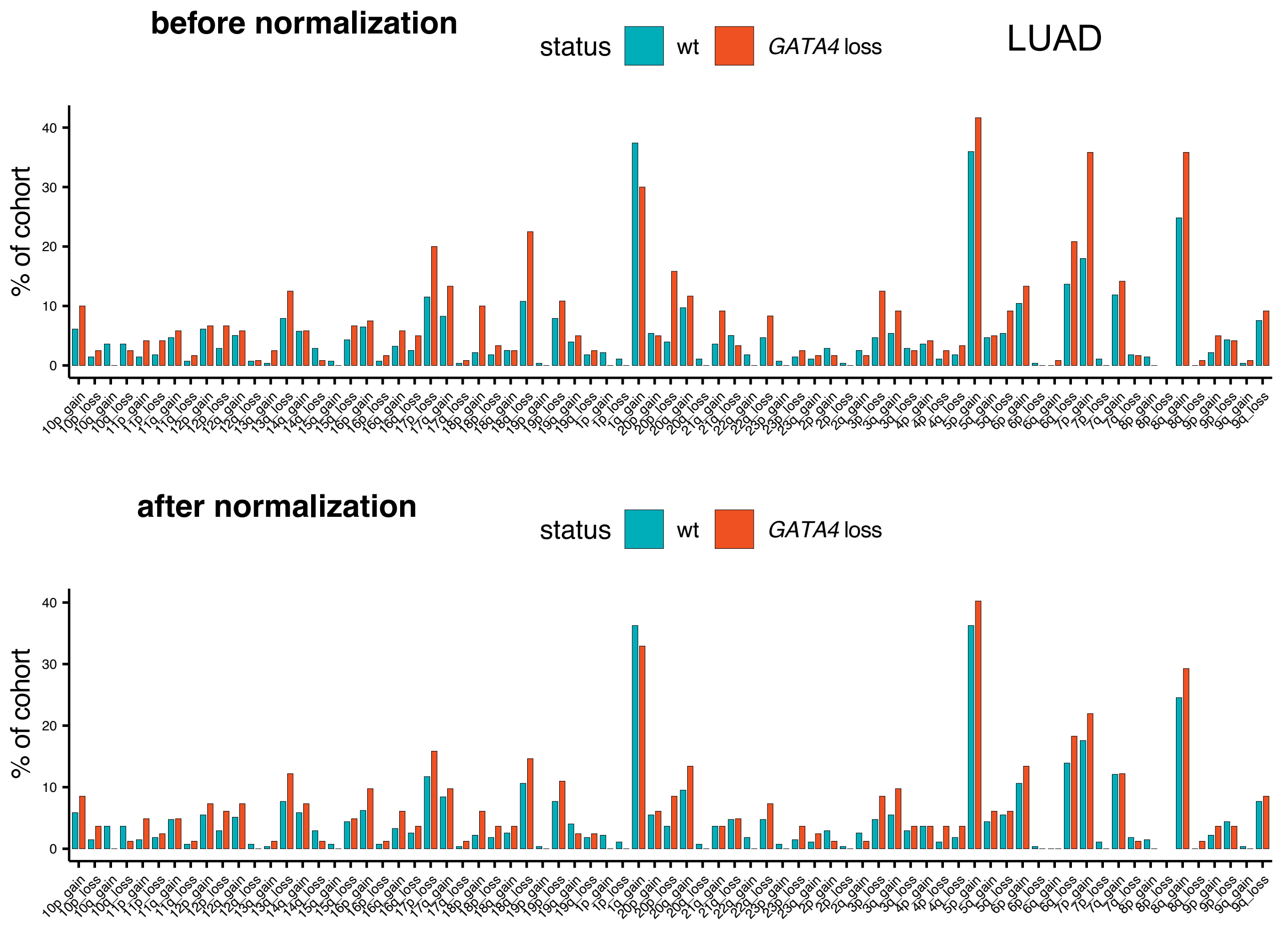
before normalization
status
wt
GATA4 loss
LUAD
% of cohort
40
30
20
10
0
after normalization
status
wt
GATA4 loss
% of cohort
40
30
20
10
0

Supplementary Figure 8 Enhanced expression of *Gata4* is tumor suppressive in multiple cell types

(A) Schematic of plasmids used to generate viruses that were transduced into murine cancer cell lines for tumor studies. The genes cloned into each of these vectors were the control, GFP, or the SASP regulator *Gata4*. Cell lines include KP lung adenocarcinoma cells derived from the autochthonous model described in Figure 1, KP lung cells transduced with a strong antigen (KP LucOS), B16 melanoma cells, and PyMT SWTP3 murine breast cancer cells. All cell lines are derived from mice of a C57BL/6 background and are thus syngeneic with C57BL/6 mice.
(B) Tumor volumes are shown for experimental time course of transplant of KP LucOS lung tumor cells in immune competent C57BL/6J mice. Dox is started one day before transplant and is administered throughout the course of the experiment. n=7 control mice, n=6 +Dox mice.
(C) Tumor volumes are shown for experimental time course of transplant of B16 melanoma tumor cells in immune competent C57BL/6J mice. Dox is started one day before transplant and is administered throughout the course of the experiment. n=6 control mice, n=5 +Dox mice.
(D) Tumor volumes are shown for experimental time course of transplant of PyMT S2WTP3 breast tumor cells in immune competent C57BL/6J mice. Dox is started one day before transplant and is administered throughout the course of the experiment. n=5 control mice, n=5 +Dox mice.
(E) KP lung adenocarcinoma cells with dox-inducible GFP or dox-inducible *Gata4* were mixed at a ratio of 2:1 (GFP:*Gata4*), and transplanted into C57BL/6 mice. All cells expressed constitutive GFP in order to control for immunogenicity. Dox was administered in the indicated group 1 day before cell transplant and is administered throughout the course of the experiment. P-value from two-tailed Mann-Whitney test and n=10 mice for each group.

Fig. S8:

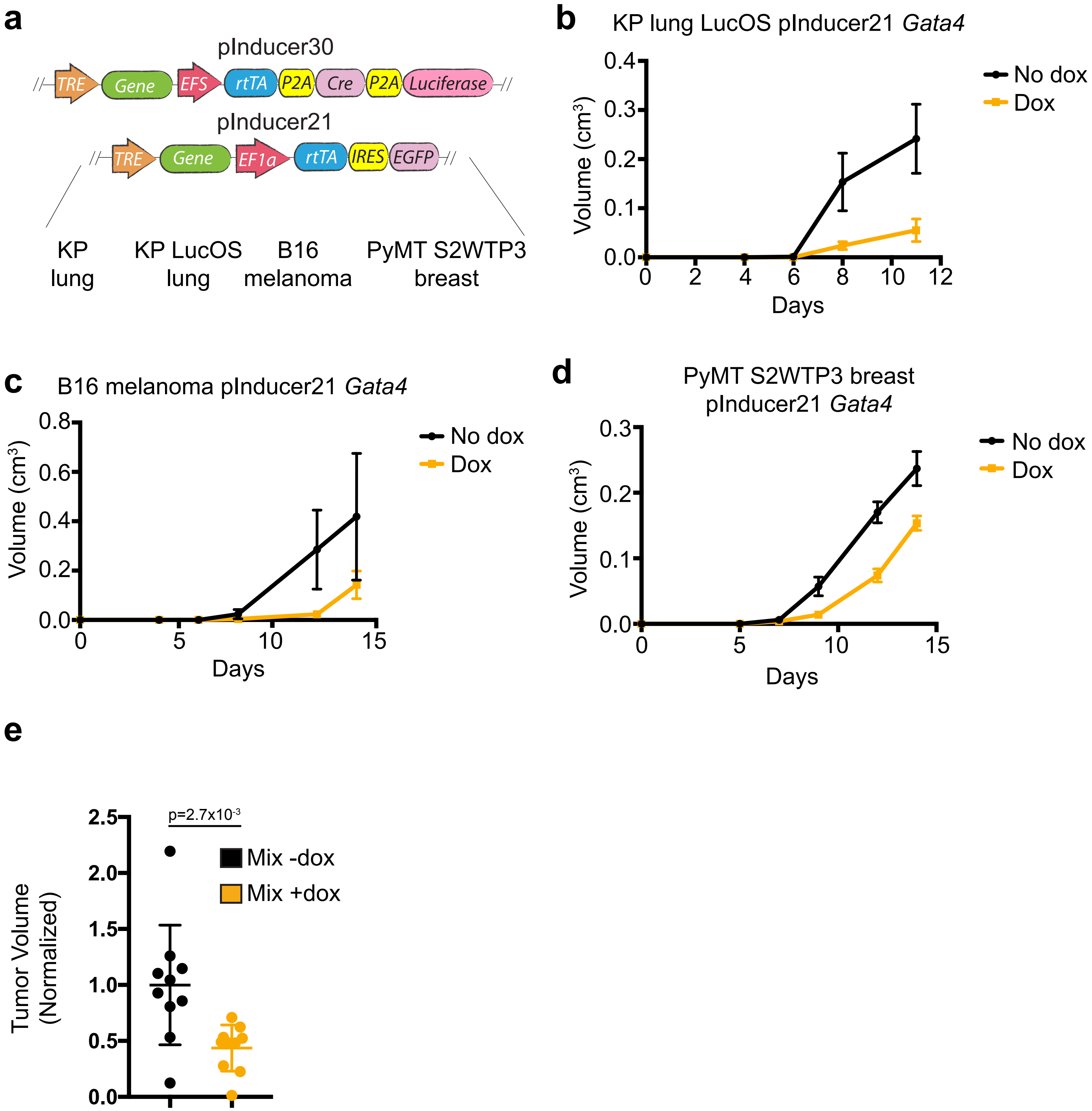

a
pInducer30
TRE
Gene
EFS
rtTA
P2A
Cre
P2A
Luciferase
pInducer21
TRE
Gene
EF1a
rtTA
IRES
EGFP
KP lung
KP LucOS lung
B16 melanoma
PyMT S2WTP3 breast
b
KP lung LucOS pInducer21 Gata4
Volume (cm³)
Days
No dox
Dox
c
B16 melanoma pInducer21 Gata4
Volume (cm³)
Days
No dox
Dox
d
PyMT S2WTP3 breast pInducer21 Gata4
Volume (cm³)
Days
No dox
Dox
e
p=2.7x10-3
Mix -dox
Mix +dox
Tumor Volume (Normalized)

Supplementary Figure 9: Antibody controls and individual tumor growth curves for experiments from Figure 4

(A) Representative flow cytometry-based analysis of CD8 (y-axis) and CD4 (x-axis) cells in the peripheral blood of mice which received the isotype control antibody.
(B) Representative flow cytometry-based analysis of CD8 (y-axis) and CD4 (x-axis) cells in the peripheral blood of mice which received the CD4 cell depleting antibody and the isotype control antibody.
(C) Representative flow cytometry-based analysis of CD8 (y-axis) and CD4 (x-axis) cells in the peripheral blood of mice which received the CD8 cell depleting antibody and the isotype control antibody.
(D) Representative flow cytometry-based analysis of CD8 (y-axis) and CD4 (x-axis) cells in the peripheral blood of mice which received both the CD4 and CD8 cell depleting antibodies.
(E) KP cells with dox-inducible *Gata4* at the specified ratios were transplanted into Rag1$^{-/-}$ C57BL/6J mice. The 33% Gata4 population was composed of 33% of cells with dox-inducible Gata4 and 67% of cells with dox-inducible GFP. Dox was administered to mice one day before tumor cell transplant and was administered continuously until endpoint. Each line represents one tumor over time. Tumor volumes are shown. Individual tumor volumes correspond with Figure 4F. n=10 per group combined from 2 independent experiments.
(F) C57BL/6J mice received depleting antibodies against CD4^{+}, CD8^{+}, or CD4^{+} and CD8^{+} T cells, or an isotype control antibody. All mice received the same total amount of antibody. The single depletion groups received isotype control antibody in addition to depletion antibody in order to ensure that all groups of mice received the same amount of antibody as the double depletion group. KP cells with dox-inducible *Gata4* were transplanted into the flanks of the pre-depleted mice. Dox was initiated one day before transplant in the indicated groups and was administered continuously until the experimental endpoint. Tumor volumes are shown. Each line represents one tumor over time. Tumor volumes correspond with Figure 4G. n=10 per group combined from 2 independent experiments.

Fig. S9:

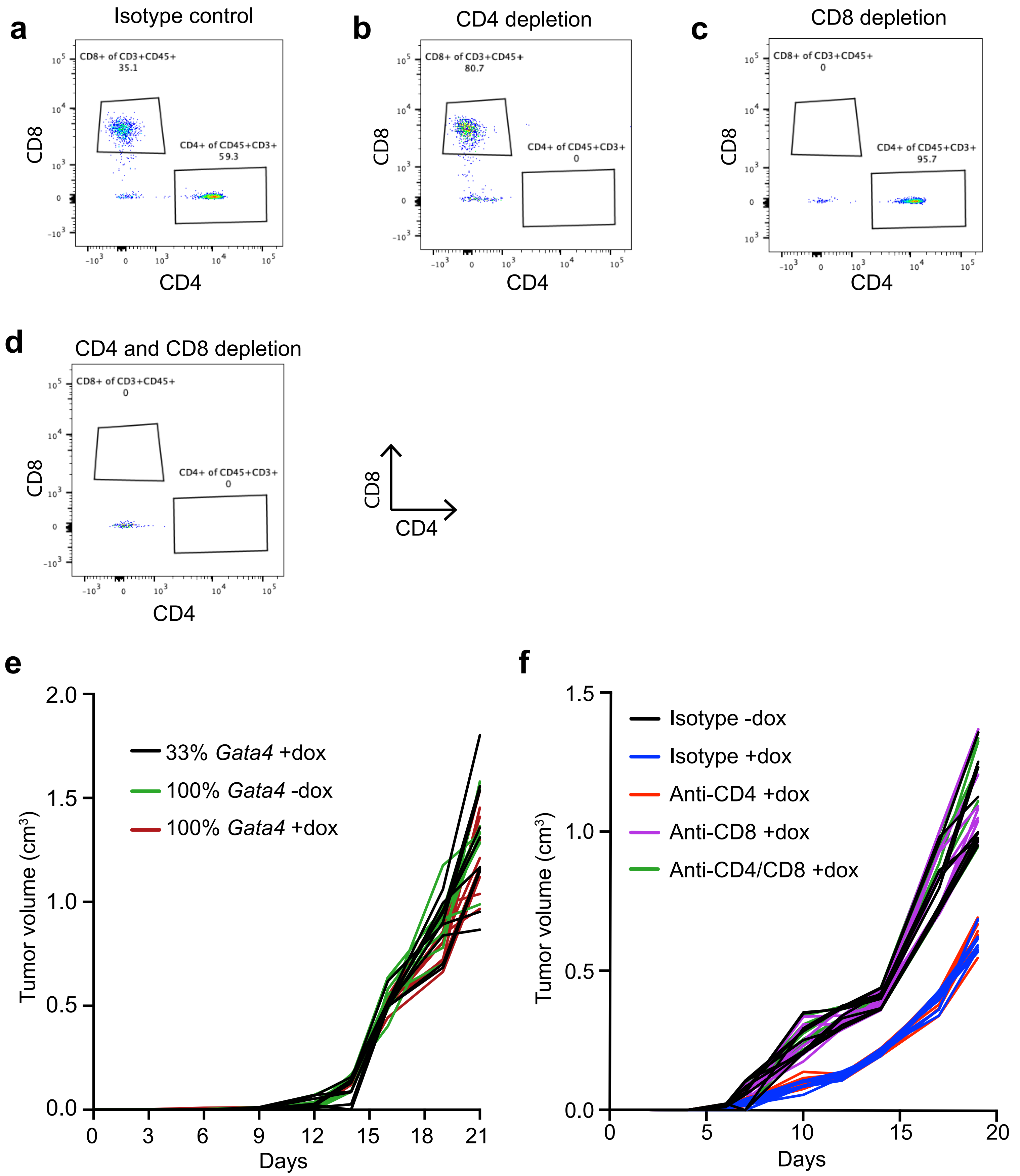
a
Isotype control
CD8+ of CD3+CD45+
35.1
CD4+ of CD45+CD3+
59.3
CD8
CD4
b
CD4 depletion
CD8+ of CD3+CD45+
80.7
CD4+ of CD45+CD3+
0
CD8
CD4
c
CD8 depletion
CD8+ of CD3+CD45+
0
CD4+ of CD45+CD3+
95.7
CD8
CD4
d
CD4 and CD8 depletion
CD8+ of CD3+CD45+
0
CD4+ of CD45+CD3+
0
CD8
CD4
CD8
CD4
e
33% Gata4 +dox
100% Gata4 -dox
100% Gata4 +dox
Tumor volume (cm3)
Days
f
Isotype -dox
Isotype +dox
Anti-CD4 +dox
Anti-CD8 +dox
Anti-CD4/CD8 +dox
Tumor volume (cm3)
Days
