# Supplementary material for: A GATA4-regulated secretory program suppresses tumors through recruitment of cytotoxic CD8 T cells
Source: Nat Commun. 2022 Jan 11;13:256. doi: 10.1038/s41467-021-27731-5 (PMC8752777; doi:10.1038/s41467-021-27731-5)
Supplement: Supplementary file 2 — Description of Additional Supplementary Files [file 41467_2021_27731_MOESM2_ESM.pdf]

Description of Additional Supplementary Files

File Name: Supplementary Data 1
Description: RNAseq data corresponding with KP autochthonous GEMM experiments described in the paper for Figure 1 and 2. First tab contains log2FC of genes in sgCtrl tumors versus sgGata4 tumors. Second tab contains log2FC of genes in sgCtrl tumors versus sgCdkn2a tumors.

File Name: Supplementary Data 2
Description: First tab contains Reactome curated pathways downregulated in sgGata4 tumors versus sgCtrl tumors from the autochthonous KP GEMM experiments. Second tab contains Reactome curated pathways upregulated in sgGata4 tumors versus sgCtrl tumors from the autochthonous KP GEMM.

File Name: Supplementary Data 3
Description: Sets of genes used to identify immune cell subtypes. First tab contains Immgen-derived gene sets. Second tab contains single cell RNAseq-derived gene sets used to identify subytpes of immune cells from RNAseq data.

File Name: Supplementary Data 4
Description: Differential expression of genes in GATA4 lost versus GATA4 not lost TCGA samples. Tabs correspond to different tumor types.

File Name: Supplementary Data 5
Description: Log2FC of genes between two groups of samples from TCGA data, as described in the text. First tab contains COAD 8p loss with 8q gain versus 8p not lost. COAD 8p loss without 8q gain versus 8p not lost. Third tab contains BRCA 8p loss with 8q gain versus 8p not lost. Fourth tab contains BRCA 8p loss without 8q gain versus 8p not lost.
