# Supplementary material for: A GATA4-regulated secretory program suppresses tumors through recruitment of cytotoxic CD8 T cells
Source: Nat Commun. 2022 Jan 11;13:256. doi: 10.1038/s41467-021-27731-5 (PMC8752777; doi:10.1038/s41467-021-27731-5)
Supplement: Supplementary file 8 — Reporting Summary [file 41467_2021_27731_MOESM8_ESM.pdf]

nature research

Corresponding author(s): Stephen J. Elledge

Last updated by author(s): Nov 16, 2021

Reporting Summary

Nature Research wishes to improve the reproducibility of the work that we publish. This form provides structure for consistency and transparency in reporting. For further information on Nature Research policies, see our Editorial Policies and the Editorial Policy Checklist.

Statistics

For all statistical analyses, confirm that the following items are present in the figure legend, table legend, main text, or Methods section.

n/a	Confirmed	
☐	☒	The exact sample size (*n*) for each experimental group/condition, given as a discrete number and unit of measurement
☐	☒	A statement on whether measurements were taken from distinct samples or whether the same sample was measured repeatedly
☐	☒	The statistical test(s) used AND whether they are one- or two-sided<br>*Only common tests should be described solely by name; describe more complex techniques in the Methods section.*
☐	☒	A description of all covariates tested
☐	☒	A description of any assumptions or corrections, such as tests of normality and adjustment for multiple comparisons
☐	☒	A full description of the statistical parameters including central tendency (e.g. means) or other basic estimates (e.g. regression coefficient) AND variation (e.g. standard deviation) or associated estimates of uncertainty (e.g. confidence intervals)
☐	☒	For null hypothesis testing, the test statistic (e.g. *F*, *t*, *r*) with confidence intervals, effect sizes, degrees of freedom and *P* value noted<br>*Give P values as exact values whenever suitable.*
☐	☒	For Bayesian analysis, information on the choice of priors and Markov chain Monte Carlo settings
☐	☒	For hierarchical and complex designs, identification of the appropriate level for tests and full reporting of outcomes
☐	☒	Estimates of effect sizes (e.g. Cohen's *d*, Pearson's *r*), indicating how they were calculated

Our web collection on statistics for biologists contains articles on many of the points above.

Software and code

Policy information about availability of computer code

Data collection	NA
Data analysis	The custom source code used to analyze some of the TCGA data are provided at the following publicly available repository: https://github.com/emmavwatson/CNorm. All other publicly available data analysis packages used in this study are described and referenced in the Methods section of the manuscript, these include: Annovar, SeqAn, Integrated Genomics Viewer, edgeR, Hisat2, featureCounts, subread, and GSEA.

For manuscripts utilizing custom algorithms or software that are central to the research but not yet described in published literature, software must be made available to editors and reviewers. We strongly encourage code deposition in a community repository (e.g. GitHub). See the Nature Research guidelines for submitting code & software for further information.

Data

Policy information about availability of data

All manuscripts must include a data availability statement. This statement should provide the following information, where applicable:

- Accession codes, unique identifiers, or web links for publicly available datasets
- A list of figures that have associated raw data
- A description of any restrictions on data availability

The RNA-Seq data are deposited in the Sequences Read Archive (SRA) with the project accession number PRJNA778768, Source data are provided with this paper. The remaining data are available within the Article, Supplementary Information or Source Data file.

Field-specific reporting

Please select the one below that is the best fit for your research. If you are not sure, read the appropriate sections before making your selection.

☒ Life sciences ☐ Behavioural & social sciences ☐ Ecological, evolutionary & environmental sciences

For a reference copy of the document with all sections, see nature.com/documents/nr-reporting-summary-flat.pdf

Life sciences study design

All studies must disclose on these points even when the disclosure is negative.

Sample size	Sample sizes were pre-determined based on feasibility of study and standard practice for the field.
Data exclusions	No data were excluded.
Replication	Replicates in the form of multiple tumors from multiple mice were analyzed for every experiment. In several cases (which we noted in the text) we performed the entire mouse experiment in duplicate.
Randomization	Randomization not relevant to study
Blinding	Blinding not relevant to study

Reporting for specific materials, systems and methods

We require information from authors about some types of materials, experimental systems and methods used in many studies. Here, indicate whether each material, system or method listed is relevant to your study. If you are not sure if a list item applies to your research, read the appropriate section before selecting a response.

Materials & experimental systems

n/a	Involved in the study	
☐	☒	Antibodies
☐	☒	Eukaryotic cell lines
☒	☐	Palaeontology and archaeology
☐	☒	Animals and other organisms
☒	☐	Human research participants
☒	☐	Clinical data
☒	☐	Dual use research of concern

Methods

n/a	Involved in the study	
☒	☐	ChIP-seq
☐	☒	Flow cytometry
☒	☐	MRI-based neuroimaging

Antibodies

Antibodies used	All antibodies used in the paper are described in the materials and methods section. The following antibodies were used for IHC: anti-phospho-Histone H3 (pHH3) (Serl0; Cell Signaling, 9701, 1:200, https://www.cellsignal.com/products/primary-antibodies/phospho-histone-h3-ser10-antibody/9701), CD3 (Abcam, ab16669, 1:200, https://www.abcam.com/cd3-antibody-sp7-ab16669.html), CD8 (Abcam, ab217344, 1:200, https://www.abcam.com/cd8-alpha-antibody-epr21769-ab217344.html), Ccl2-neutralizing antibody (#BE0185; BioXCell, 100 ug/100 uL, https://bxcell.com/product/m-r-h-ccl2-mcp-1/), Anti-PD-1 antibody (#BP0273, BioXCell, https://bxcell.com/product/invivoplus-anti-mouse-pd-1-cd279/).
Validation	All antibodies were purchased commercially from the company specified in the materials and methods for each antibody and authenticated by the manufacturer as described on their websites.

Eukaryotic cell lines

Policy information about cell lines

Cell line source(s)	Cell lines were purchased from ATCC; B16 murine melanoma cells & PyMT S2WTP3 murine breast cancer cells
Authentication	Cell lines that were purchased from ATCC were previously authenticated by the ATCC using STR analysis, according to the product specification.
Mycoplasma contamination	Cell lines were tested for mycoplasma and were negative.
Commonly misidentified lines (See ICLAC register)	No commonly misidentified lines were used in this paper.

Animals and other organisms

Policy information about studies involving animals; ARRIVE guidelines recommended for reporting animal research

Laboratory animals	Laboratory animal information and details are provided in the methods or in the figure legends and also in the text, depending on the experiment. GEMM model: KrasLSL-G12D/+; p53fl/fl; Rosa26LSL-Cas9 (KPC) mice. KPC mice were maintained on a mixed C57BL/6:SV129 genetic background. C57BL/6 mice and CD57BL/6 Rag1-/- mice were acquired from Jackson Labs and were of both sexes.
Wild animals	No wild animals were used for this study.
Field-collected samples	No field-collected samples were used in the study.
Ethics oversight	All animal studies described in this study were approved by the Brigham & Women's Hospital Institutional Animal Care and Use Committee or the MIT Institutional Animal Care and Use Committee ethical guidelines.

Note that full information on the approval of the study protocol must also be provided in the manuscript.

Flow Cytometry

Plots

Confirm that:

☒ The axis labels state the marker and fluorochrome used (e.g. CD4-FITC).

☒ The axis scales are clearly visible. Include numbers along axes only for bottom left plot of group (a 'group' is an analysis of identical markers).

☒ All plots are contour plots with outliers or pseudocolor plots.

☒ A numerical value for number of cells or percentage (with statistics) is provided.

Methodology

Sample preparation	Samples were described in the paper text and methods. For RNA-Seq Tumor tissues were frozen in RNAlater solution (Thermo #PAS-39542) and RNA was extracted using the RNeasy plus mini kit (Qiagen #74134). cDNA libraries were built using NEB Next Ultra RNA Library Prep Kit for lllumina (NEB #E7530S). Samples were multiplexed using NEB Next Multiplex Oligos for lllumina (NEB #E7710L).<br>For Antibody depletion CD4-depleting antibody (#BP0003-1), CDS-depleting antibody (#BP0061), and the isotype control antibody (#BP0090) were acquired from BioXCell. Antibodies were diluted using dilution buffer (BioXCell #IP0070) to 100 ug/100 uL. 200ug of the relevant antibody mixture (l00ug CD4-depleting antibody plus 100 ug isotype control antibody, l00ug CDS-depleting antibody plus l00ug isotype control antibody, 200 ug isotype control antibody, or 100 ug CD4-depleting antibody plus 100 ug CDS-depleting antibody) were given to each mouse 1 day before tumor cell transplant and every 3 days subsequently. Circulating lymphocytes were profiled by collecting peripheral blood and flow cytometric analysis. For lmmunohistochemistry<br>Mice were euthanized by carbon dioxide asphyxiation. Lungs were perfused through the trachea with 4% paraformaldehyde (PFA), fixed overnight, transferred to 70% ethanol and subsequently embedded in paraffin. Sections were cut at a thickness of four micrometers and stained with H&E for pathological examination. Chromogenic immunohistochemistry (IHC) was performed on a Ventana Medical Systems Discovery XT instrument with online deparaffinization using Ventana's reagents and detection kits and antigen retrieved in Ventana Cell Conditioner 1 or 2.
Instrument	The instrument used was a BD LSRII.
Software	FacsDIVA was used to collect the data and FlowJo was used for all flow cytometry data analysis.
Cell population abundance	Information about cell population abundance is in the paper and in the methods.
Gating strategy	Gating strategies were utilized according to standard practice for flow cytometry.

☒ Tick this box to confirm that a figure exemplifying the gating strategy is provided in the Supplementary Information.
